# Metabolic regulation of endothelial senescence

**DOI:** 10.3389/fcvm.2023.1232681

**Published:** 2023-08-15

**Authors:** Nhat-Tu Le

**Affiliations:** Center for Cardiovascular Regeneration, Department of Cardiovascular Sciences, Houston Methodist Research Institute, Houston, TX, United States

**Keywords:** cellular metabolism, cellular senescence, endothelial cells, atherosclerosis, SASP

## Abstract

Endothelial cell (EC) senescence is increasingly recognized as a significant contributor to the development of vascular dysfunction and age-related disorders and diseases, including cancer and cardiovascular diseases (CVD). The regulation of cellular senescence is known to be influenced by cellular metabolism. While extensive research has been conducted on the metabolic regulation of senescence in other cells such as cancer cells and fibroblasts, our understanding of the metabolic regulation of EC senescence remains limited. The specific metabolic changes that drive EC senescence are yet to be fully elucidated. The objective of this review is to provide an overview of the intricate interplay between cellular metabolism and senescence, with a particular emphasis on recent advancements in understanding the metabolic changes preceding cellular senescence. I will summarize the current knowledge on the metabolic regulation of EC senescence, aiming to offer insights into the underlying mechanisms and future research directions.

## 1. Introduction

As individuals grow older, their susceptibility to various disorders and diseases, including CVD and cancer, tends to increase. These conditions share common molecular mechanisms, such as cellular senescence and chronic inflammation, which contribute to their pathogenesis (1, 2). CVD remains the leading cause of morbidity and mortality worldwide, especially among individuals aged 65 and above. Several scoring systems have been developed to assess the risk of CVD, including the Pooled Cohort Equations, Framingham Risk Score, and Reynolds Risk Score, with age being a significant risk factor for both females and males (1–3). The hallmarks of aging encompass diverse biological factors that influence the aging process at the cellular and molecular levels. These hallmarks, which include genomic instability, telomere attrition, epigenetic alterations, loss of proteostasis, deregulated nutrient sensing, mitochondrial dysfunction, cellular senescence, and altered intercellular communication, are interconnected, and can mutually influence each other. Collectively, they contribute to the process of aging and the development of age-related diseases (4–6). In their review, Viscomi and Zeviani (2019) emphasize the essential role of well-functioning mitochondria in cellular function and overall well-being. The authors highlight the critical roles of energy generation through oxidative phosphorylation (OXPHOS) and the implications of mitochondrial dysfunction in the pathology of age-related diseases. They discuss various factors that contribute to maintaining mitochondrial health, including proper nutrition, regular exercise, and minimizing exposure to environmental toxins. Furthermore, the authors propose potential therapeutic approaches, such as the use of antioxidants and lifestyle interventions, aimed at promoting mitochondrial health and mitigating the impact of mitochondrial dysfunction (7).

Numerous studies consistently demonstrate that restricted food intake can extend the lifespan of various species (8–12). The association between restricted food intake and increased lifespan in laboratory rats dates back to the 1930s (13), and subsequent studies have confirmed this finding. Restricting food intake by 30–60 percent in mice and rats leads to similar increases in average and maximal lifespan. However, it is important to note that rats with nearly unrestricted caloric intake, despite being lean and engaging in exercise, exhibit an increase in average lifespan but not maximal lifespan (13–19). Conversely, obesity is a known risk factor for various age-related diseases and is associated with a shorter lifespan. Therefore, adopting a healthy diet and increasing physical activity can be beneficial in preventing the onset of age-related conditions. Interestingly, individuals who possess exceptional health characteristics share similarities with adult volunteers who have followed caloric restriction regimens (20–22). These findings highlight the importance of preventing malnutrition and reducing overall caloric intake, rather than focusing on specific nutrients, to derive the benefits of caloric restriction on lifespan. Moreover, these findings emphasize the significant role of cellular metabolism in regulating essential cellular processes such as proliferation, survival, and senescence (23).

Adenosine triphosphate (ATP), the primary energy currency in cells, is predominantly generated through mitochondrial OXPHOS in the presence of oxygen. However, under certain conditions, such as cellular senescence, ATP production can be altered, and alternative pathways such as anaerobic glycolysis may become more prominent (24). While ECs are typically quiescent in adults, they can be activated in response to various internal and external stimuli (25–28). Extensive evidence has illuminated the crucial role of dysfunctional senescent ECs in vascular dysfunction, making EC senescence a key factor in age-related diseases, particularly CVD. Given the pivotal role of ECs in human health and disease, comprehending the metabolic changes associated with EC senescence is imperative for unraveling the mechanisms involved in age-related CVD.

## 2. Key processes in cellular metabolism

Cellular metabolism constitutes an intricate network of biochemical reactions that convert nutrients into ATP, the essential energy source for various cellular functions, including proliferation, response to stimuli, and maintenance of structure and function (29). The link between cellular metabolism and cellular activity is exemplified by the interplay between immune cell metabolism and their functionality. Undernutrition can lead to immunosuppression and increased vulnerability to infection and autoimmune diseases. Conversely, overnutrition can result in chronic low-grade inflammation, elevating the risk of metabolic disorders and CVD (30, 31). Another illustrative example is the exploitation of host cell metabolism by viruses to optimize viral production, often resembling the metabolic changes observed in cancer cells. This exploitation involves heightened nutrient consumption and anabolism to support replication or rapid cultivation of the virus (32). Key processes in cellular metabolism include glycolysis [including gluconeogenesis and pentose phosphate pathway (PPP)] (33–35), OXPHOS (36), glutamine metabolism (37), and FAO (38–40). Glycolysis serves as the initial step in cellular metabolism, converting glucose into pyruvate (33–35). OXPHOS operates through the electron transport chain (ETC) to generate ATP and harness energy (36). Glutamine metabolism involves the utilization of the non-essential amino acid (NEAA) glutamine for ATP production and biosynthetic purposes (37). FAO entails the breakdown of fatty acids to generate ATP (38–40). Collectively, these processes maintain cellular energy balance and contribute to optimal cellular function.

### 2.1. Glycolysis

**Figure 1:**
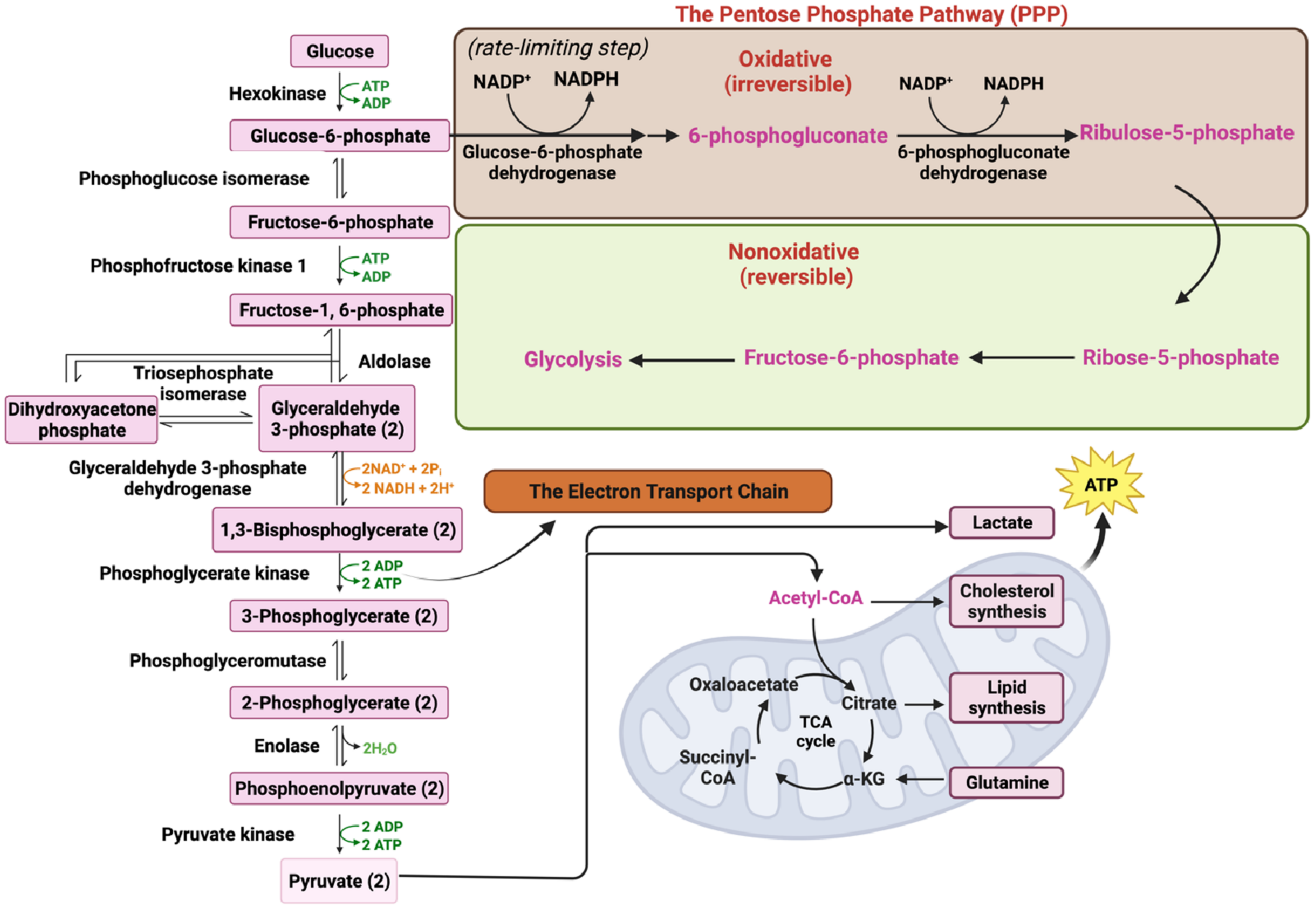
Key processes in cellular metabolism (figure created using BioRender).

Cells acquire extracellular glucose through glucose transporters (GLUTs), primarily GLUT1 and GLUT4, which belong to the solute carrier family 2. This glucose uptake initiates glycolysis, a complex metabolic pathway involving various enzymes. The enzymes involved in glycolysis include hexokinase, phosphoglucose isomerase, phosphofructokinase-1, triosephosphate isomerase, glyceraldehyde 3-phosphate dehydrogenase, phosphoglycerate kinase, phosphoglyceromutase, enolase, and pyruvate kinase (PK) (Figure 1) (35, 41, 42).

Glycolysis begins with the conversion of fructose 6-phosphate to fructose 1,6-bisphosphate (FBP) catalyzed by the aldolase enzyme. FBP plays a crucial role not only in glycolysis but also in gluconeogenesis and the PPP (Figure 1) (43, 44). Gluconeogenesis is the process of *de novo* glucose synthesis from available precursors, which plays a crucial role in maintaining glucose homeostasis to meet energy demands, particularly during prolonged starvation in animals (45). The PPP branches from glucose 6-phosphate, generating Nicotinamide Adenine Dinucleotide Phosphate (NADPH) and ribose 5-phosphate while also redirecting carbons back to the glycolytic or gluconeogenic pathway (Figure 1) (46).

**Figure 2:**
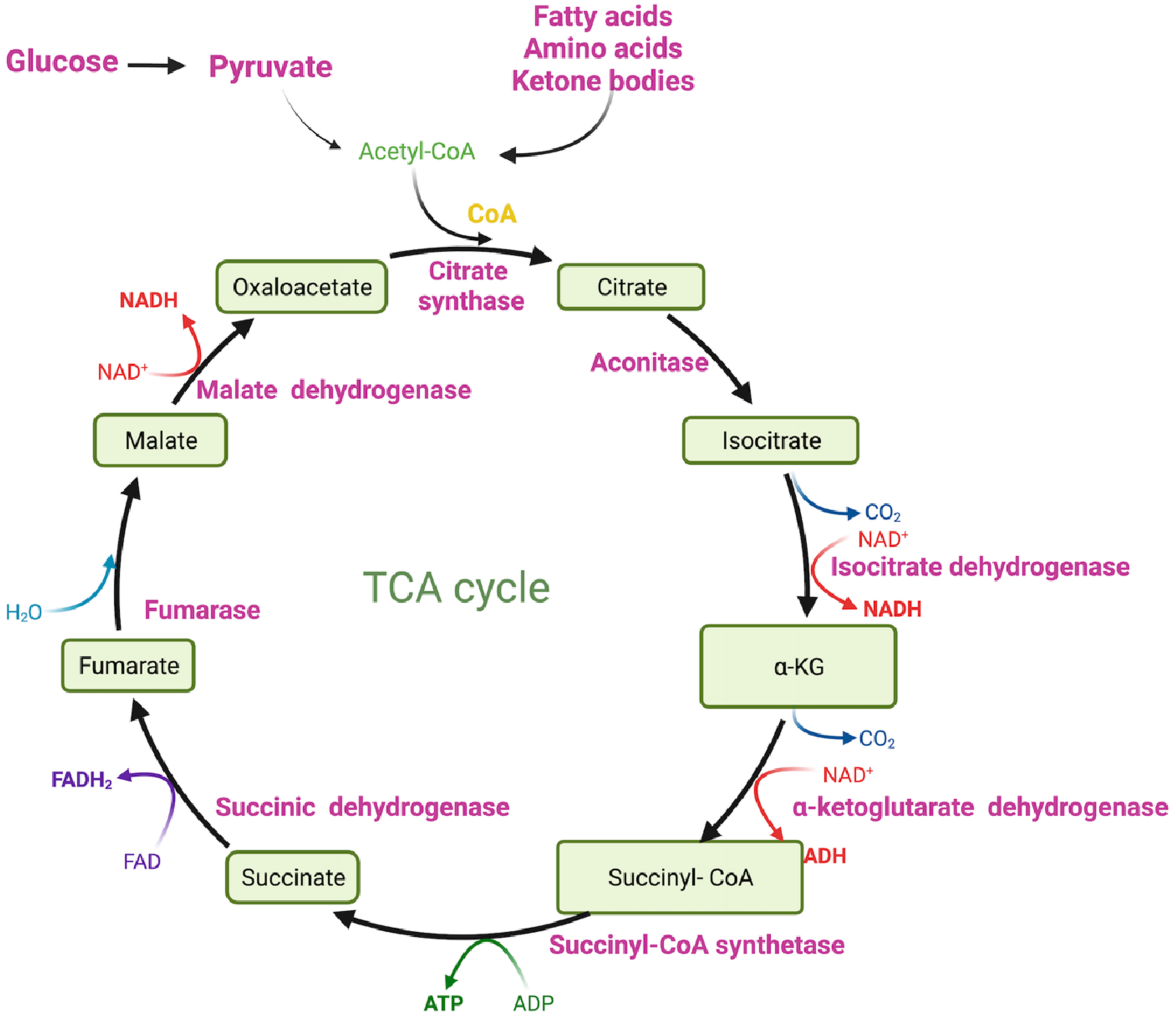
The TCA cycle (figure created using BioRender).

Ultimately, glucose is converted to pyruvate through the action of the enzyme PK. Pyruvate can undergo further metabolism, either by being converted to lactate via lactate dehydrogenase (LDH) (Figure 1) or by serving as a substrate in the tricarboxylic acid (TCA) cycle to generate ATP (Figures 1, 2) (33, 34). The TCA cycle, also referred to as the Krebs cycle or the citric acid cycle, is a pivotal metabolic pathway that takes place in the mitochondria of cells. In the TCA cycle, citrate undergoes conversion to isocitrate (isocitric acid, ICA) through the action of the enzyme aconitase. ICA serves as a significant intermediate not only in energy metabolism but also as a precursor for the biosynthesis of amino acids and fatty acids. Moreover, ICA exhibits antioxidant properties that contribute to cellular protection against oxidative stress (47, 48). Another intermediate in the TCA cycle, cis-aconitic acid, is formed from ICA by the enzyme aconitase and then converted back to ICA through the action of aconitate hydratase. This step is crucial for ATP generation through the oxidation of acetyl-CoA in the TCA cycle (Figure 2) (47, 48).

**Figure 3:**
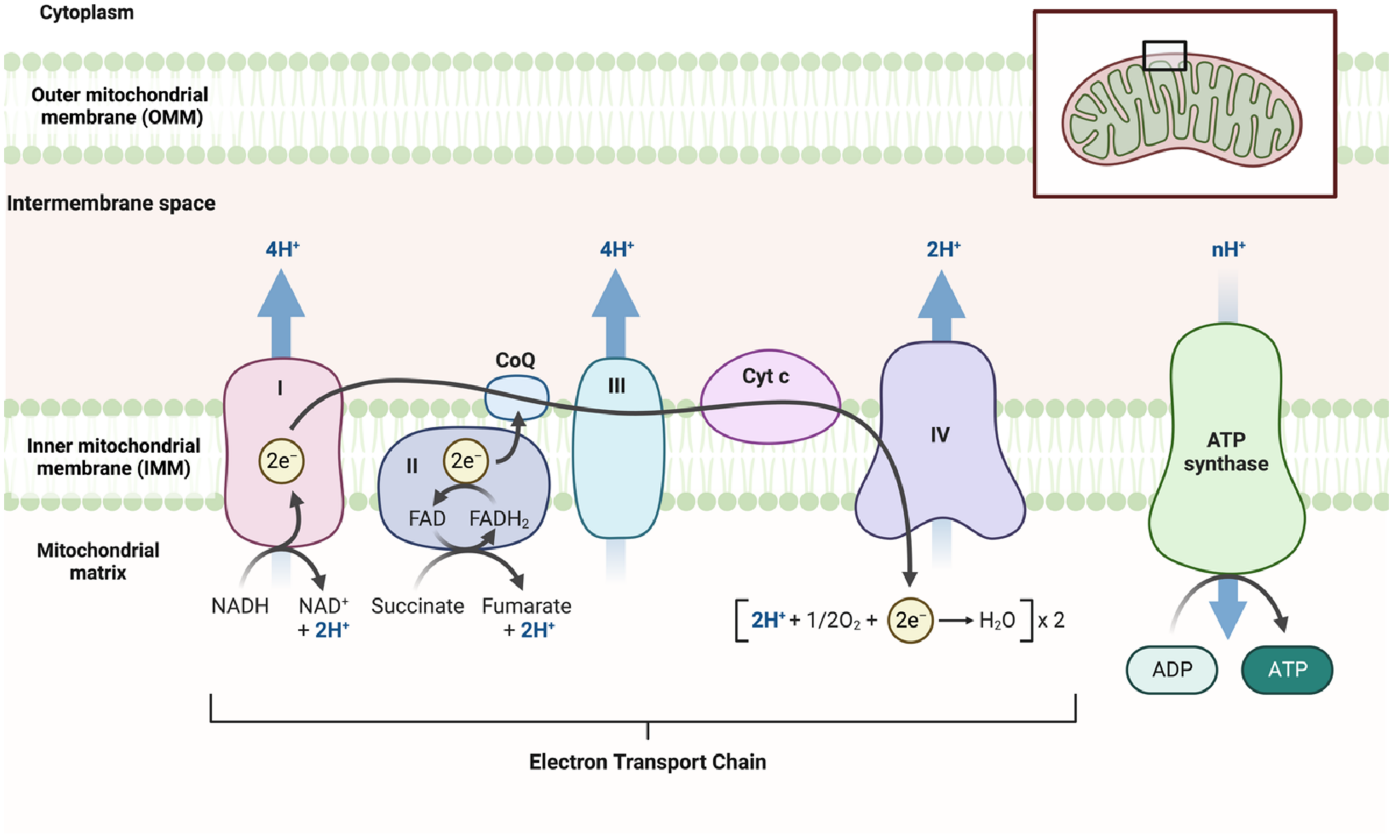
The electron transport chain (figure created using BioRender).

During glycolysis, Nicotinamide Adenine Dinucleotide+ (NAD^+^) is reduced to its active form, Nicotinamide Adenine Dinucleotide + hydrogen (NADH) (Figure 1), which is subsequently oxidized in the ETC to generate ATP (Figure 3). NADH also plays a crucial role in other energy-related processes such as the TCA cycle and OXPHOS (Figures 2, 3) (49–51).

### 2.2. OXPHOS

In addition to its role in the TCA cycle (Figure 2), pyruvate can also serve as a fuel for OXPHOS, a metabolic process that also takes place in the mitochondria and leads to ATP generation. OXPHOS involves a series of reactions within the ETC, which locates in the mitochondria. These ETC reactions establish a proton gradient across the inner mitochondrial membrane (IMM), and the energy derived from this gradient is utilized by ATP synthase to generate ATP (Figure 3) (36).

**Figure 4:**
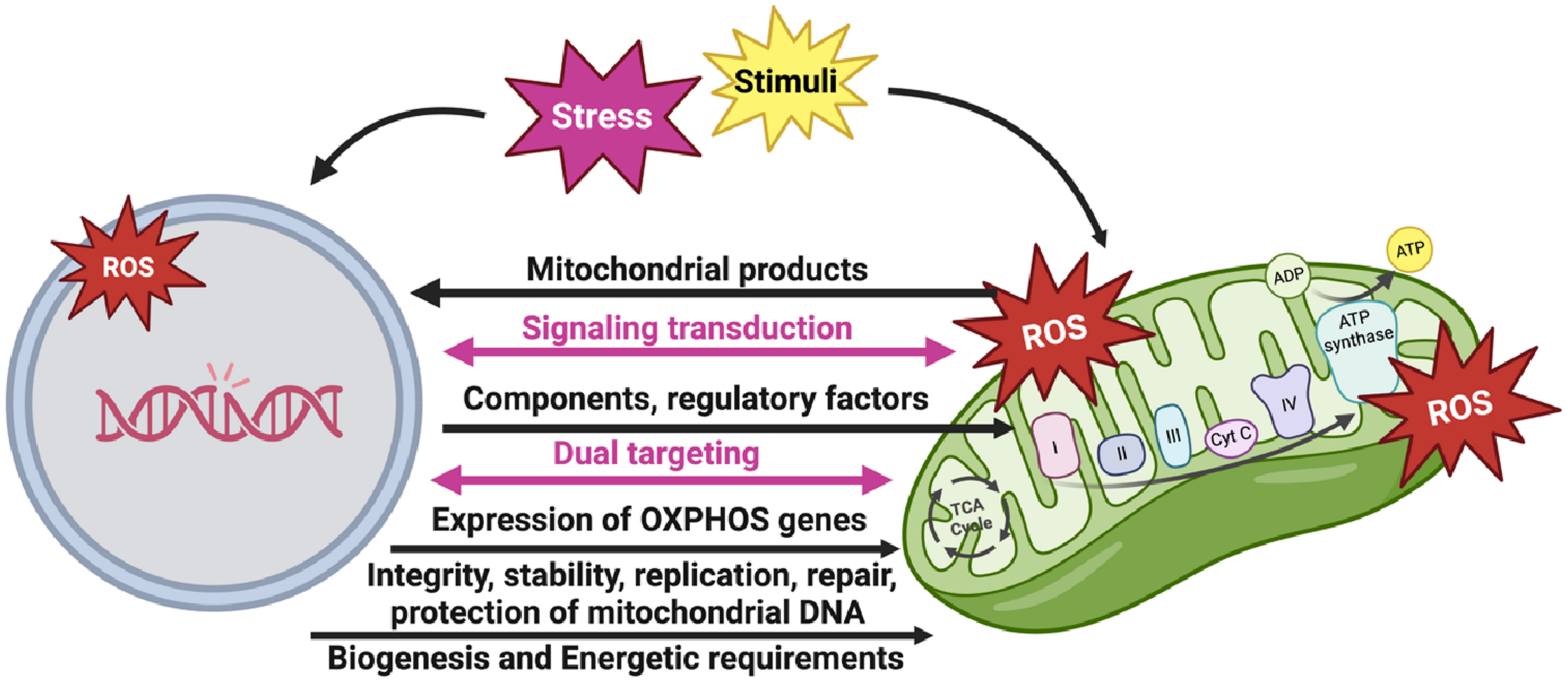
The nucleus-mitochondria communication (figure created using BioRender).

It is worth noting that the interaction and coordination between the nucleus and mitochondria play a crucial role in the formation of the OXPHOS system (Figure 4). This system consists of protein complexes located within the IMM. While the nucleus provides most of the necessary components and regulatory factors for OXPHOS assembly, the mitochondrial DNA (mtDNA) encodes vital subunits of the OXPHOS complexes. This coordinated process allows for efficient synthesis, assembly, regulation, and maintenance of the OXPHOS complexes, enabling cellular energy production and metabolic adaptation.

The nuclear genome plays a pivotal role in controlling the expression of OXPHOS genes in response to cellular and environmental conditions. This allows cells to adjust their energy production based on changing energy demands, metabolic states, and environmental stresses. Nuclear transcription factors can respond to nutrient availability, oxygen levels, and cellular energy status, thereby modulating the expression of nuclear-encoded OXPHOS genes and fine-tuning OXPHOS activity.

Furthermore, the nuclear genome is responsible for preserving the integrity and stability of mtDNA, which encodes some OXPHOS subunits. Nuclear-encoded factors participate in mtDNA replication, repair, and protection against damage. Proper maintenance of mtDNA ensures the continuous production of functional mitochondria and preserves OXPHOS capacity.

Nuclear-encoded subunits are synthesized in the cytoplasm and then imported into mitochondria, where they combine with mitochondria-encoded subunits. The nuclear genome encodes critical factors that facilitate the import, assembly, and stability of mitochondria-encoded subunits, ensuring the proper formation and function of the OXPHOS complexes (Figure 3) (52).

### 2.3. Glutamin metabolism

**Figure 5:**
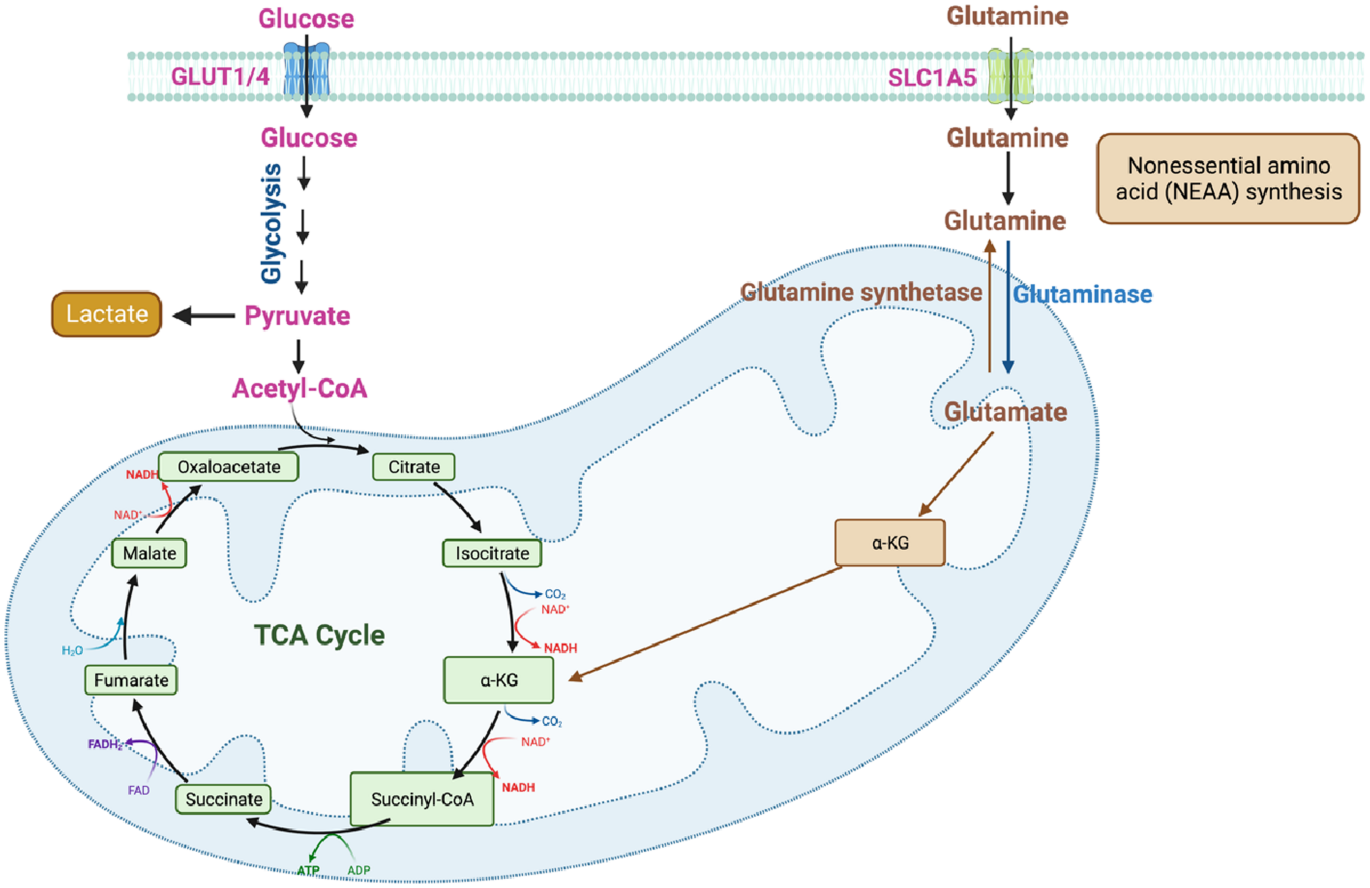
Glutamine metabolism (figure created using BioRender).

Glutamine, a non-essential amino acid (NEAA), plays a vital role in cellular metabolism, participating in multiple metabolic pathways. It is involved in protein and nucleotide biosynthesis, as well as the synthesis of other amino acids. Glutamine serves as a significant substrate for ATP production and can also be converted into glucose through gluconeogenesis in the liver. Additionally, it contributes to the regulation of acid-base balance, immune response, and oxidative stress (Figure 5). Within the body, glutamine undergoes enzymatic reactions to convert into glutamate, which can further be metabolized in the TCA cycle or converted into other metabolites such as ammonia and α-ketoglutarate (αKG) (Figures 1, 5). The metabolism of glutamine is tightly regulated to maintain a balance between biosynthesis, energy production, and other essential physiological processes. Disruptions in glutamine metabolism have been implicated in various diseases, including cancer, metabolic disorders, and neurological disorders (37).

### 2.4. FAO

FAO is a metabolic process in which fatty acids are broken down into acetyl-CoA, which can then enter the TCA cycle to generate ATP (Figure 2). FAO takes place in various organelles, including the mitochondria (beta-oxidation), peroxisome (alpha- and beta-oxidation), and endoplasmic reticulum (omega-oxidation). It serves as a significant source of energy during fasting periods and high energy demand, such as exercise. During FAO, circulating mediators like epinephrine and glucagon increase the rate of lipolysis, releasing fatty acids from adipose tissue. These fatty acids can then be utilized for energy production through FAO. Tissues such as skeletal muscle, heart muscle, and kidneys heavily rely on FAO for energy production when glycogen and gluconeogenic precursors are limited. FAO provides an alternative, highly efficient mode of energy production while preserving muscle tissue from catabolic breakdown. Therefore, FAO plays a vital role in maintaining energy homeostasis within the body (38–40).

Glycolysis and FAO are two major metabolic pathways involved in energy generation. The utilization of these pathways depends on cellular compartmentation, cell type, energy demands, and substrate availability. Glycolysis takes place in the cytoplasm and is a rapid process that provides ATP production while supplying metabolites for biosynthetic pathways. It utilizes glucose or other sugars as substrates and can occur in the presence or absence of oxygen, known as aerobic or anaerobic conditions, respectively. Glycolysis yields a net of 2 ATP molecules per glucose molecule and involves enzymes such as hexokinase and phosphofructokinase. The end products of glycolysis, such as pyruvate, can enter the mitochondria for further energy production through OXPHOS or be converted to lactate in anaerobic conditions. On the other hand, FAO takes place in the mitochondria and relies on fatty acids derived from dietary fats or stored triglycerides as substrates. FAO requires oxygen, specifically aerobic conditions, and generates a significant amount of ATP. The key enzyme involved in FAO is carnitine palmitoyltransferase 1 (CPT1), which facilitates the transport of fatty acids into the mitochondria for OXPHOS. The end product of FAO is acetyl-CoA, which enters the TCA cycle for further ATP production. FAO is highly efficient in generating ATP from stored fats and serves as the primary energy source during prolonged fasting or endurance activities. Cells can dynamically switch between glycolysis and FAO to adapt to changing metabolic requirements and environmental conditions. This flexibility allows cells to effectively meet their energy needs and maintain energy homeostasis (53–56).

## 3. Cellular senescence

### 3.1. Different mechanisms of cellular senescence

Cellular senescence, a well-recognized hallmark of aging, has emerged as a promising target for preventing various age-related diseases including cancer and cardiovascular conditions such as atherosclerosis, acute myocardial infarction, cardiac aging, pressure overload-induced hypertrophy, heart regeneration, hypertension, and abdominal aortic aneurysm. The mechanisms underlying cellular senescence are intricate, as it can have both beneficial and detrimental effects in cancer and cardiovascular conditions, depending on specific circumstances (57, 58). The groundbreaking work of Hayflick and Moorhead in 1961 provided valuable insights into cellular senescence. Their observations revealed that human diploid fibroblasts undergo irreversible cell cycle arrest after a certain number of divisions (59–62). This arrest occurs when telomeres reach a critical length known as the Hayflick limit (4, 5, 63, 64), impairing the formation of t-loops and ultimately leading to cellular senescence (59–62).

**Figure 6:**
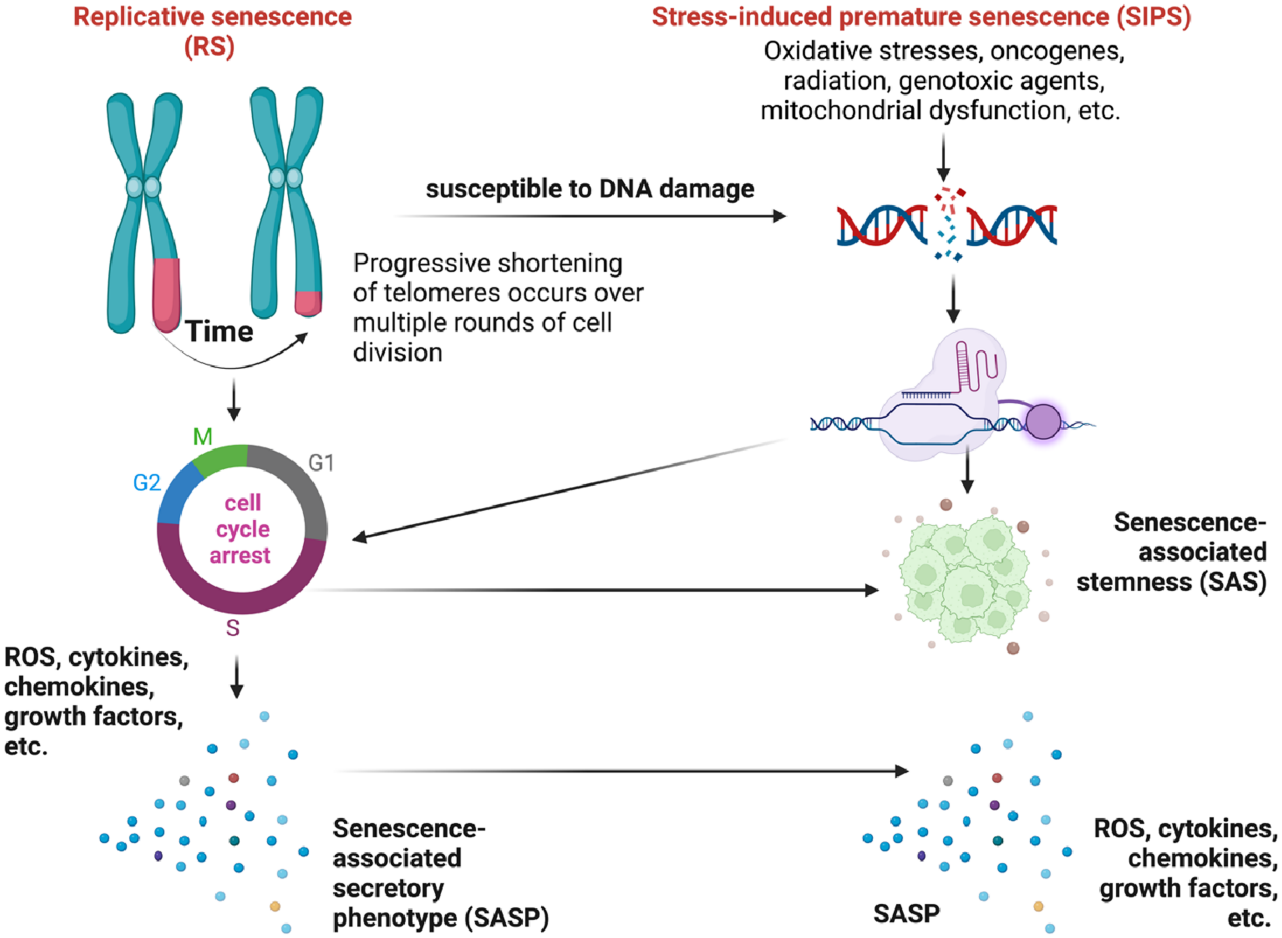
Different mechanisms of cellular senescence (figure created using BioRender).

Telomeres act as protective caps at the ends of chromosomes composed of repetitive hexanucleotide sequences (5′-TTAGGG-3′). They play a crucial role in maintaining genomic stability (65–67). Throughout each round of DNA replication, telomeres undergo a DNA damage response (DDR) due to their similarity to damaged DNA (59–62, 68–71). Although telomerase, the enzyme that maintains telomeres, can add telomeric repeats to chromosomal ends (72), many human somatic cells lack telomerase activity. As a result, with each cell division, telomeres progressively shorten as the DNA strands cannot be fully replicated (73–75). Upon reaching the Hayflick limit, telomeres trigger a DDR signal, leading to cellular senescence and cell cycle arrest (59–62, 65–67) and is characterized by the upregulation of senescence-associated markers such as senescence-associated β-galactosidase (SA-β-gal), cyclin-dependent kinase inhibitors p21^Cip1/Waf1^ and p16^INK4a^. Cellular senescence can disrupt tissue homeostasis and increase susceptibility to age-related disorders and diseases, including cardiovascular conditions and cancer. This specific form of growth arrest was later coined replicative senescence (RS) (Figure 6) (69–71). RS is a gradual process that occurs over time as cells undergo repeated replication cycles. During this process, senescent cells have been observed to accumulate exponentially with increasing chronological age in multiple tissues (76, 77). Studies have shown that individuals over the age of 60 with shorter telomeres tend to have lower survival rates compared to younger individuals (78).

The relationship between telomere shortening and cellular senescence is a complex phenomenon (79–82), as cellular senescence can occur independently of telomere shortening (72). The association between telomere length and certain age-related conditions such as cardiomyocyte hypertrophy and fibrosis is limited (83). Interestingly, it has been observed that cells expressing telomerase can still undergo senescence despite having long telomeres (72, 84). In the context of cardiovascular conditions, telomere shortening in leukocytes is primarily attributed to telomere attrition over time rather than inherent short telomere length at birth (85). Furthermore, cellular senescence can also be triggered prematurely by chronic stress through various stimuli, independent of telomere shortening. This phenomenon is referred to as stress-induced premature senescence (SIPS) (86–90). These stressors can originate from both internal and external factors, including oncogenes, radiation, cancer treatments, mitochondrial dysfunction, ROS, and more (illustrated in Figure 6). SIPS is characterized by the upregulation of the p53/p21^Cip1/Waf1^, p16I^NK4a^/pRB pathways, positive staining for SA-β-gal, and, in some cases, telomere shortening (77). Unlike RS, SIPS can occur even in the absence of significant telomere shortening, as we (63, 64, 91) and others (77, 92) have discussed previously.

Senescent cells, including both RS and SIPS (Figure 6), secrete a diverse array of molecules collectively known as the senescence-associated secretory phenotype (SASP). The SASP is composed of soluble proteins and extracellular vesicles (EVs), which include pro-inflammatory cytokines, chemokines, growth factors, pro-angiogenic factors, small molecules, lipids (such as nitric oxide (NO) and prostaglandin E2 (PGE2)), ROS, and proteases (90, 93, 94), which collectively contribute to chronic inflammation and the manifestation of senescence-associated phenotypes (59–64, 90, 91, 93–110). It is worth noting that the SASP can exhibit reversibility under certain conditions (94, 102, 103, 105, 107, 111–114). SIPS can be induced in various cell types when exposed to oxidative stress or specific substances. For example, hydrogen peroxide or Ultraviolet B exposure can induce SIPS in immortalized human foreskin fibroblasts expressing telomerase (hTERT-BJ1) (60). Another example involves the induction of SIPS in renal tubular cells exposed to the urine of patients with calcium oxalate kidney stones, which is likely a result of oxidative stress induced by oxalate and calcium oxalate monohydrate (115). These examples highlight the significant role of oxidative stress as a major contributor to the induction of cellular senescence through the SIPS pathway (116).

Under normal circumstances, the factors comprising the SASP facilitate communication between senescent cells and neighboring cells, including immune cells, through paracrine or autocrine signaling (63, 64, 91). This intercellular communication plays a vital role in the recruitment of immune cells such as T cells, macrophages, and natural killer cells, facilitating the efficient removal of senescent cells. This clearance process is essential for maintaining tissue homeostasis. However, during the aging process, the immune response may become impaired, resulting in compromised clearance of senescent cells (77, 92). This phenomenon, known as “immunosenescence” (117), can contribute to the accumulation of senescent cells and their associated effects. Immunosenescence has a profound impact on innate and adaptive immunity, and one notable example of its pathogenic potential in cardiovascular diseases is the production of proinflammatory cytokines and chemokines by senescent T cells (118). Both genotoxic chemotherapy agents and radiation therapy have been found to induce mitochondrial dysfunction and cellular senescence (63). To investigate this further, we conducted a study involving 16 thoracic cancer patients who underwent radiation therapy. We collected peripheral blood mononuclear cells (PBMCs) before and three months after radiation therapy and employed mass cytometry to characterize human immune cell lineages. We examined markers of senescence, DDR, efferocytosis, and determinants of clonal hematopoiesis of indeterminate potential (CHIP). Our analysis revealed a decrease in the frequency of B cell subtypes after radiation therapy. Clustering of the mass cytometry data allowed us to identify 138 functional subsets of PBMCs. Upon comparing the post-mass cytometry samples with the baseline, we observed an increased expression of TBX21 (T-bet) in the largest subset of Ki67–/DNMT3a+ naive B cells. Notably, T-bet expression correlated with p90RSK phosphorylation. Additionally, we observed elevated CD38 expression in naive B cells (CD27–) and CD8+ effector memory CD45RA T cells (TEMRA). *In vitro* experiments confirmed the significant role of p90RSK activation in upregulating CD38+/T-bet+ memory and naive B cells, as well as myeloid cells. We also observed increased β-galactosidase staining and mtROS following radiation exposure. Collectively, these findings suggest that p90RSK activation plays a pivotal role in immunosenescence (119).

Senescent cells release EVs that contain specific molecules such as miRNAs, long non-coding RNAs, and proteins (120). EVs, including exosomes and microvesicles, play diverse biological roles as carriers of proteins, nucleic acids, and metabolites between cells, influencing the behavior of recipient cells (120–126). Notably, activated senescent cells have been observed to generate a higher quantity of functional EVs compared to non-senescent cells, primarily due to the upregulation of p53 expression. These EVs have the capability to enhance the production of ROS and promote senescence in neighboring cells. Moreover, EVs contribute to cellular senescence in recipient cells by transporting factors that induce senescence (127, 128).

Senescent cells remain metabolically active. For example, cultured human diploid fibroblasts in a senescent state express higher levels of enzymes involved in the glycolytic pathway, such as hexokinase, phosphoglycerate kinase, and phosphoglycerate mutase, resulting in increased glycolytic activity compared to young fibroblasts (129, 130). Additionally, senescent cells exhibit upregulation of mtROS production and succinate induction, even when both OXPHOS and glycolysis are inhibited by low dose ionizing radiation, without undergoing necrosis or apoptosis (131).

### 3.2. Mitochondrial dysfunction and cellular senescence

Mitochondrial dysfunction, recognized as one of the nine “hallmarks” of aging (5), highlights the connection between cellular metabolism and senescence (7, 132). As the aging process progresses, there is a decline in respiratory capacity and mitochondrial membrane potential, often accompanied by an augmented production of free radicals (133). The intricate interplay between aging and mitochondrial dysfunction establishes a detrimental cycle that contributes to the development of a senescent phenotype (133, 134).

ATP is primarily produced through glucose metabolism, which involves the breakdown of glucose via glycolysis (43–46). Glucose is converted into pyruvate, which undergoes further metabolism through processes such as lactate metabolism, the TCA cycle, and OXPHOS (33, 34, 49–51). OXPHOS occurs through the ETC, which consists of two mobile electron carriers (ubiquinone and cytochrome c) and four multi-subunit enzyme complexes located in the IMM (Figure 3). These complexes are known as complex I (NADH ubiquinone oxidoreductase), complex II (succinate dehydrogenase), dimeric complex III (cytochrome bc1 oxidoreductase), and complex IV (cytochrome c oxidase) (135). NADH and flavin adenine dinucleotide (FADH2), generated in reactions such as the TCA cycle or FAO, donate electrons to the ETC, leading to ATP production (136). During ETC activity, mtROS are generated, including superoxide anions, hydroxyl radicals, peroxyl radicals, and other species capable of generating free radicals, particularly from complexes I to III1 (136).

Mitochondrial ROS (mtROS) plays a crucial role in initiating various signaling pathways and is necessary for maintaining normal cellular function (137). Additionally, mtROS contributes significantly to the persistent SASP induced by various stimuli, including cancer treatments (131). During the aging process, decreased antioxidant production leads to elevated levels of ROS, resulting in the oxidation of lipids, proteins, and DNA (138). When DNA is exposed to ROS, guanine (G) in the DNA can undergo oxidation and modification, resulting in the formation of 8-oxo guanine (8-oxoG). 8-oxoG can base-pair with cytosine (C) or adenine (A). Consequently, during subsequent rounds of replication, DNA polymerase may erroneously insert an A opposite 8-oxoG instead of C. This can lead to the insertion of thymine (T) opposite A, resulting in a C>A substitution and ultimately causing genomic instability (139, 140). Importantly, mtDNA is more susceptible to DNA damage induced by ROS compared to nuclear DNA due to less efficient DNA repair mechanisms (132). Therefore, mitochondrial dysfunction-mediated mtDNA damage also plays a crucial role in the development of pathological conditions, including age-related.

## 4. Metabolic regulation of cellular senescence

### 4.1. Metabolic regulation of cellular biomass

**Figure 7:**
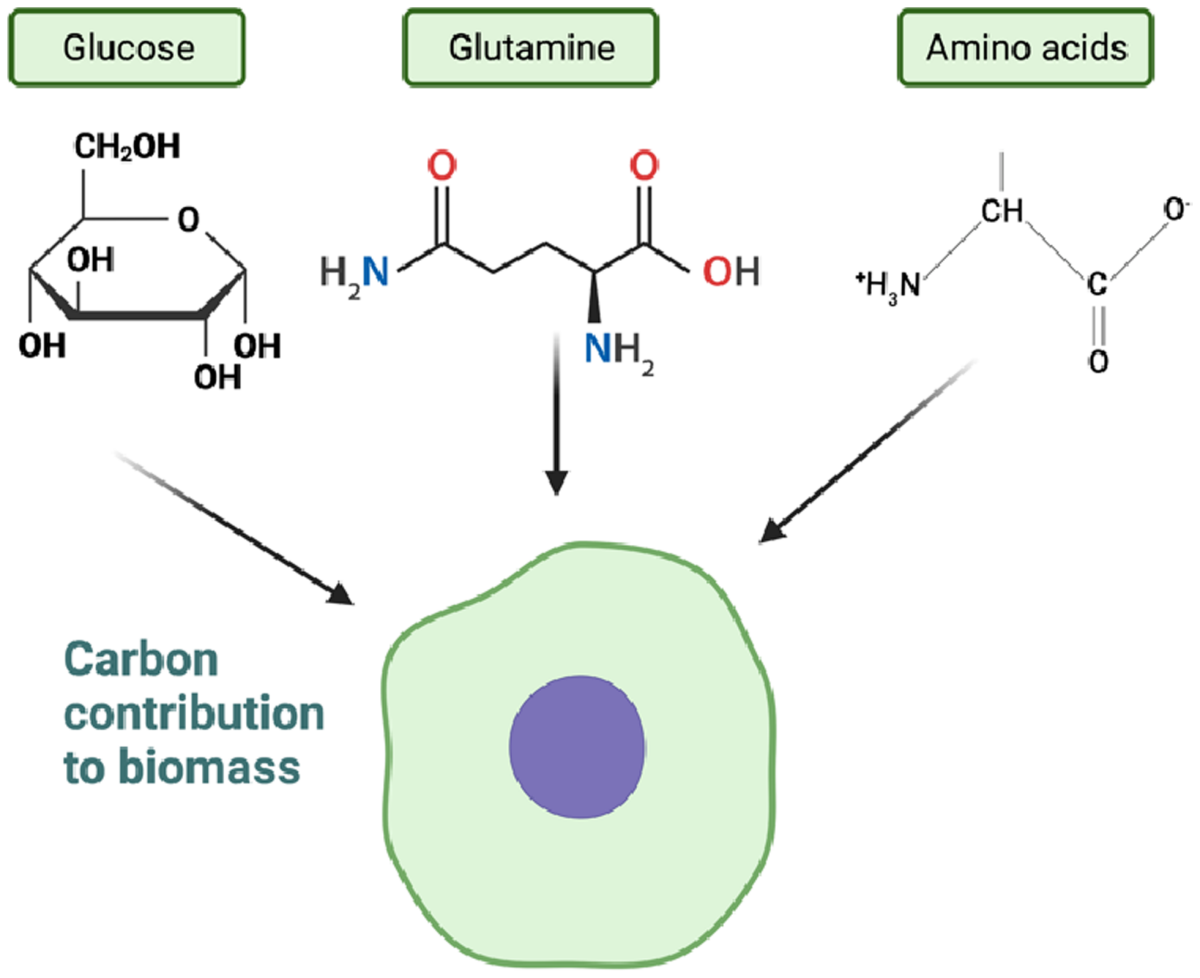
Carbon contribution to biomass (figure created using BioRender).

While cells utilize various nutrients such as glucose and glutamine through cellular metabolism to support growth and proliferation, they also secrete waste metabolites such as lactate and glutamate, which contribute to cellular biomass. Although glucose and glutamine are extensively consumed nutrients, their carbon contribution to cellular biomass is relatively small compared to other amino acids. Other amino acids play a more significant role in determining cell mass (Figure 7). The exact source of approximately 40% of the remaining carbon has not been definitively identified, but lipids, particularly palmitate and oleate, contribute to a portion of this carbon pool (141–148). The regulation of cellular biomass involves bidirectional feedback mechanisms that control the rate of cell growth and division (141–147). These mechanisms, including the PI3K-AKT-mTOR and RAS-MAPK pathways, play a crucial role in controlling the rate of cell growth. The comprehensive review by Mikael Bjorklund (2019) highlights the importance of these mechanisms in cell size sensing and regulation (148).

Kim et al. (2010) conducted a study to investigate the mechanisms underlying the increase in cellular biomass during cellular senescence, employing organelle-specific fluorescence dyes. Their findings revealed a progressive enlargement of membranous organelles accompanied by the upregulation of lipogenic enzymes, including fatty acid synthase (FAS), ATP citrate lyase (ACLY), and acetyl-CoA carboxylase. This upregulation resulted in the accumulation of membrane lipids. They also observed an elevation in the mature form of sterol regulatory element-binding protein (SREBP)-1, a transcription factor involved in lipogenesis. The expression of these lipogenic effectors was found to be higher in the liver tissues of aging Fischer 344 rats. Furthermore, the researchers demonstrated that ectopic expression of mature SREBP-1 in Chang cells and young human dermal fibroblasts (HDFs) was sufficient to induce senescence. Inhibition of lipogenesis using FAS inhibitors and siRNA-mediated silencing of SREBP-1 and ACLY significantly attenuated H2O2-induced senescence. Notably, treatment with FAS inhibitors successfully reversed the aging phenotype of HDFs, restoring them to a more youthful state. These findings highlight the occurrence of enhanced lipogenesis, driven by the induction of SREBP-1, as a common feature in cellular senescence. The lipogenic process contributes to the enlargement of organelles and overall cell mass observed during senescence (149).

Cellular senescence is characterized not only by the enlargement of membranous organelles such as the endoplasmic reticulum and the Golgi apparatus but also by an increase in lysosome mass. This increase is linked to enhanced lysosomal biogenesis and activity, which play a role in the induction of the SASP (150–152). Additionally, a study conducted by Correia-Melo et al. (2016) demonstrated that senescent human fibroblasts exhibit increased mitochondrial mass and membrane potential compared to young cells. The researchers found that inhibiting mitochondrial biogenesis through siRNA targeting PGC-1α, a key regulator of mitochondrial biogenesis, led to decreased production of ROS and reduced expression of senescence markers. These findings suggest that the expansion of mitochondrial mass contributes to cellular senescence (153, 154).

Cellular biomass exhibits significant variation among individual cells and across generations. In 2018, Philipp Thomas developed a framework to predict cell size statistics within a lineage tree of a proliferating population. This approach enables the characterization of size distributions at different levels, including cell size snapshots, distribution within a population tree, and distribution of lineages across the tree. The study reveals substantial disparities in these size distributions compared to observations of isolated single cells. Within populations, cells tend to grow to different sizes, accompanied by reduced cell-to-cell variability and distinct sensitivities to cell cycle noise and division errors. Recent single-cell data supports these findings and provides further insights into the implications for maintaining a narrow size distribution (155). Studies utilizing single-cell analysis have demonstrated that animal cell growth during the cell cycle does not follow a simple linear or exponential pattern, indicating the need for accurate and realistic models. Although technical procedures have become more precise, interpreting experimental results remains a major challenge in understanding the metabolic regulation of cell size. This challenge is evident in the extrapolation of linear growth observed in S phase and the existence of multiple models that can equally fit high-quality data. Furthermore, single-cell studies have shown that despite significant variability in growth rates at the individual cell level, cells maintain a stable size distribution and a more predictable average growth rate at the population level. Interestingly, the comparison between the “adder” and “sizer” models suggests that maintaining size homeostasis requires larger cells to grow slower relative to their size than smaller cells. The mechanisms through which cells sense their size to modulate their growth rates are still poorly understood and likely more complex than initially anticipated. It is crucial to elucidate how different signaling pathways, particularly those involved in metabolism, contribute to the regulation of cell size. For example, while manipulating mTOR activity can alter cell size, this kinase does not appear to be a key player in cell-size sensing in animal cells. Additionally, mTOR activity does not seem to be involved in setting cell size-dependent mitochondrial activity, whereas the mevalonate/cholesterol pathway has been implicated. On the other hand, the p38 MAPK pathway has been linked to reducing cell size variability while coordinating cell size and cell cycle progression. Reconciling these diverse findings presents a challenge. Given the role of CDK4 inhibitors in breast cancer, further investigation into the role of CDK4 in regulating the target size of cells is necessary, as it may directly link metabolic regulation of cell size to cancer (141–148).

### 4.2. A shift towards a glycolytic phenotype preceding the changes associated with RS

**Figure 8:**
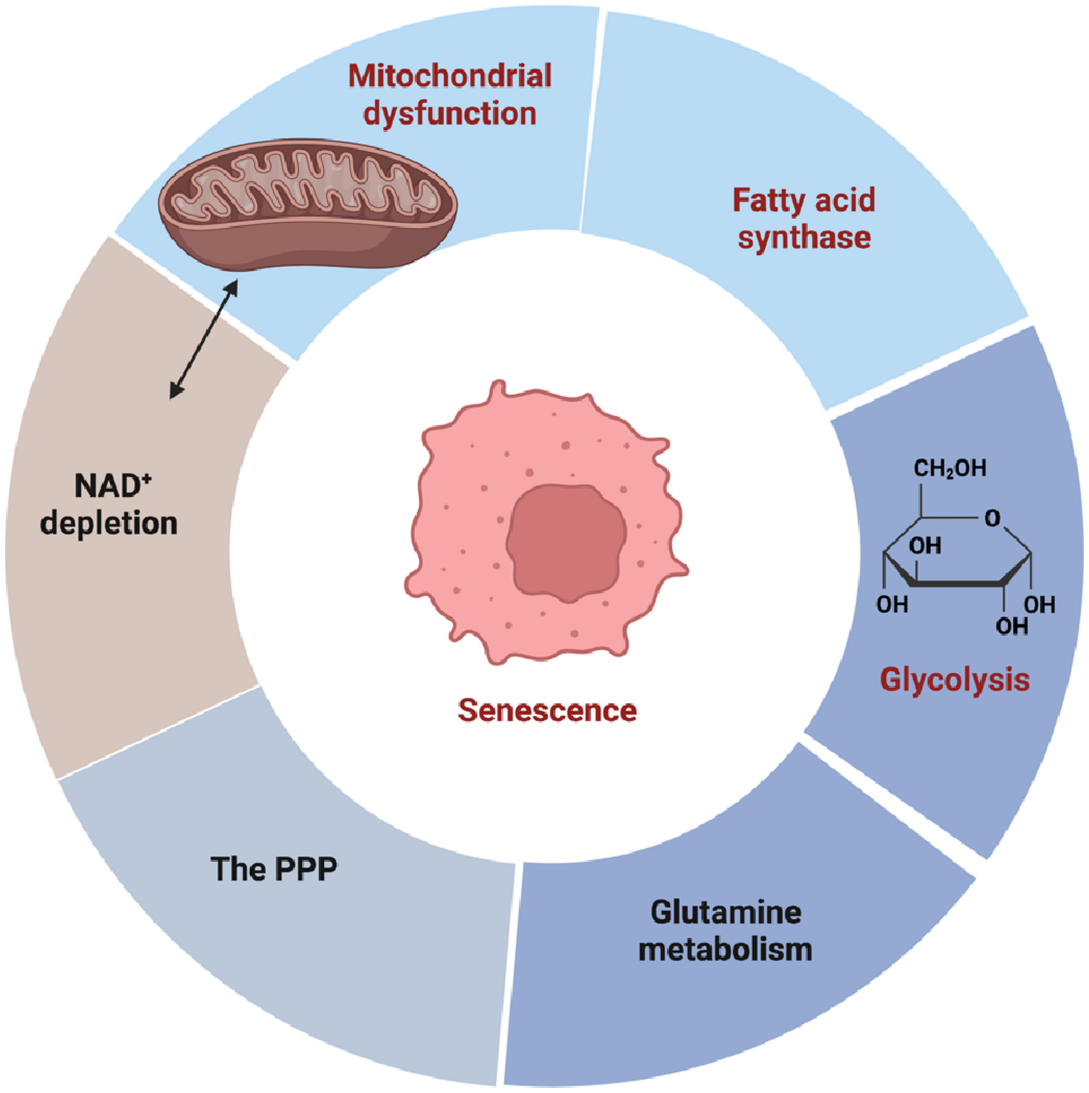
The metabolic origin of cellular senescence (figure created using BioRender).

There is compelling evidence indicating the involvement of metabolic changes in the aging process and cellular senescence (Figure 8) (156–158). Almost 40 years ago, Bittles and Harper (1984) conducted groundbreaking research revealing a metabolic shift towards glycolysis in replicative senescent HDFs. This shift was characterized by increased glucose consumption and lactate production. The dysregulation of glycolytic enzyme activity accompanied this metabolic alteration, resulting in reduced ATP and GTP levels as cells entered RS. Importantly, this metabolic alteration occurred before the typical morphological changes associated with RS, indicating its early onset (130). Subsequent studies consistently observed a shift towards glycolysis in senescent cells cultured *in vitro*. For example, Zwerschke et al. (2003) investigated metabolic changes in young and senescent HDFs and found that senescent cells exhibited increased glucose, pyruvate, and serine consumption, as well as elevated lactate, alanine, and glutamate production. However, there was no significant change in glutamine consumption. These findings highlight the transition to glycolysis preceding the changes associated with RS (156). In the senescent state, dysregulation of glycolytic enzymes leads to ATP depletion and a significant increase in cellular ADP and AMP levels, which, in turn, induces SIPS. Studies have shown that the addition of exogenous mononucleotides, such as AMP, to the culture medium can induce senescence (159). However, further investigation is needed to fully understand the metabolic alterations in SIPS. It is worth noting that the extent of this metabolic shift may vary among different cell types, and exceptions to this general mechanism exist. For example, senescent human mammary epithelial cells that do not exhibit increased glucose consumption and lactate secretion. Considering the multifaceted role of pyruvate in various metabolic process, including glycolysis, OXPHOS, and the TCA cycle, targeting the pyruvate hub has emerged as a promising therapeutic strategy for several diseases, such as diabetes, ischemic heart disease, and cancer. However, it is important to recognize that the fate of pyruvate can differ depending on the stimuli inducing cellular senescence (157, 158, 160, 161). Therefore, further research is needed to fully comprehend the specific metabolic alterations associated with different stimuli of cellular senescence. By gaining a deeper understanding of these metabolic changes, we can potentially uncover novel therapeutic avenues for combating cellular senescence and its associated pathologies.

James et al. (2015) conducted an analysis of conditioned media from senescent fibroblasts and identified several markers indicating increased glycolysis (24). These findings further support the association between senescence and the metabolic shift towards glycolysis, highlighting its potential significance in inducing cellular senescence. In a murine lymphoma model of chemotherapy-induced senescence (therapy-induced senescence, TIS), Dorr et al. (2013) demonstrated that blocking glucose consumption resulted in tumor regression and improved treatment outcomes (162). This effect is likely attributed to the elimination of senescent cancer cells and their SASP, which can promote inflammation and the proliferation of cancer cells evading TIS. These findings underscore the involvement of metabolic changes in the acquisition of the SASP during cellular senescence and the functional connection between a highly glycolytic state and senescent phenotypes (157).

However, the relationship between cellular metabolism and senescence is highly influenced by the specific cell type, the cellular context, and the stimuli triggering senescence. For instance, in a study conducted by Kotla et al. (2021), researchers made the intriguing observation that senescent myeloid cells exhibit elevated levels of mtROS and succinate, even when both OXPHOS and glycolysis are inhibited (131). These findings suggest the involvement of alternative metabolic pathways or mechanisms that contribute to the metabolic alterations observed in senescence. Nevertheless, a comprehensive understanding of the intricate and multifaceted interplay between cellular metabolism and senescence necessitates further investigation. Continued research in this area will provide valuable insights into this complex relationship and shed light on potential therapeutic strategies targeting cellular metabolism in senescence.

### 4.3. Fatty acid synthase (FASN) activity initiates the induction of cellular senescence

Lipids are vital macromolecules that perform diverse biological functions, including maintaining the integrity of cellular membranes, serving as a source of energy storage, and acting as signaling molecules (163). Changes in lipid composition and levels can significantly impact cellular function and overall physiological state. Recent studies have revealed the crucial role of specific lipids in the regulation of senescence, with some lipids even contributing to the low-grade inflammation associated with the SASP (164). One key enzyme involved in lipid metabolism is FASN, which plays a critical role in lipogenesis by controlling the synthesis of fatty acids from malonyl-CoA and acetyl-CoA. These precursors are derived from pyruvate, produced through the process of glycolysis. Fafián-Labora et al. (2019) conducted a study demonstrating that inhibiting FASN in both mouse and human cells effectively prevents the induction of cellular senescence by reducing mitochondrial bioenergetics (165, 166). Inhibition of FASN activity has been shown to impede p53-mediated senescence induction and the secretion of key factors associated with the SASP, such as IL-1α, IL-1β, and IL-6. Additionally, FASN inhibition reduces the release of EVs that propagate pro-senescence signals in a paracrine manner (165, 167). These findings highlight the involvement of FASN in driving the senescent phenotype and the associated pro-senescence signaling. However, it is worth noting that Ford (2010) conducted a study demonstrating that p53 activation inhibits FASN, indicating the presence of a negative feedback loop (168). These findings uncover the complex regulatory mechanisms involving FASN and p53 in the context of senescence, and further research is necessary to fully comprehend their interplay and implications in cellular senescence.

### 4.4. Reciprocal regulation of p53 and malic enzyme (ME) in metabolic regulation of cellular senescence

ME is an enzyme associated with the TCA cycle that catalyzes the decarboxylation of malate to pyruvate, generating either NADPH or NADH depending on the specific isoform involved. NADPH serves as a source of reducing equivalents for anabolic processes and can be regenerated through the “malate oxidation shunt” or PPP (169). Two isoforms of ME, ME1 and ME2, play a critical role in maintaining redox homeostasis in senescent cells. Both ME1 and ME2 are involved in NADPH production, lipogenesis, and glutamine metabolism, with ME2 having a more significant impact (170, 171).

Cellular senescence serves as both a protective mechanism against cancer and a contributor to aging in multicellular organisms. Increasing evidence supports the impact of metabolic changes on cell fate decisions and tumor suppression mediated by p53, the tumor suppressor protein with a crucial role in inducing and maintaining senescence. Jiang et al. (2013) conducted a study demonstrating that p53 represses the expression of ME1 and ME2. By inhibiting these enzymes, p53 regulates cellular metabolism and proliferation. The downregulation of ME1 and ME2 leads to the reciprocal activation of p53 through distinct mechanisms mediated by Mdm2 and AMPK, establishing a feed-forward loop that reinforces p53 activation. Interestingly, the downregulation of ME1 and ME2 modulates the outcome of p53 activation, resulting in a robust induction of senescence rather than apoptosis. Conversely, the enforced expression of ME suppresses senescence. These findings provide insights into the physiological functions of MEs, uncover a positive feedback mechanism that sustains p53 activation, and highlight the connection between cellular metabolism and senescence mediated by p53. Additionally, during aging, the expression of malate dehydrogenase (MDH)1, a mitochondrial enzyme involved in malate oxidation to oxaloacetate, as well as MDH2, the cytosolic enzyme of the malate-aspartate shuttle, declines (172). This leads to impaired transfer of reducing equivalents into the mitochondria. Consequently, the decreased cytosolic NAD^+^/NADH ratio in HDFs is associated with the induction of RS (173). These observations underscore the importance of ME and malate metabolism in maintaining redox balance and cellular homeostasis during the aging process. Further research is needed to elucidate the precise molecular mechanisms underlying the regulation and functional consequences of ME in cellular senescence (169).

### 4.5. The PPP in cellular senescence

The PPP has been extensively investigated in the context of cellular senescence. Although senescent cells exhibit reduced demand for deoxyribonucleotides (dNTPs), they still require NADPH to support the activity of enzymes involved in detoxifying ROS, such as thioredoxins, glutaredoxins, and peroxiredoxins. Furthermore, RS can be triggered by a shortage of dNTPs caused by decreased substrate availability. During senescence, the activity of glucose-6-phosphate dehydrogenase (G6PDH), the initial enzyme of the oxidative branch of the PPP, is diminished. Consequently, G6PDH-deficient cells experience accelerated senescence when exposed to oxidants. However, this process can be partially rescued by introducing telomerase through ectopic expression, suggesting a connection between telomerase activity and the PPP. Interestingly, transgenic mice overexpressing G6PDH have been found to exhibit an extended lifespan, which has been attributed to increased NADPH levels. Furthermore, reducing the expression of the tumor suppressor gene ataxia-telangiectasia mutated (ATM) can restore glucose flux throughout the PPP, enabling cells to overcome senescence (174–177). These studies underscore the significance of the PPP in providing NADPH and maintaining redox balance during cellular senescence. Further investigations are needed to fully comprehend the regulatory mechanisms and functional implications of the PPP in senescent cells, which may have implications for understanding the aging process and developing interventions to modulate senescence-associated pathways.

### 4.6. Glutamine metabolism induces cellular senescence

Glutamine plays a critical role in glutamine-dependent anaplerosis, a process that supplies αKG to the TCA cycle upon DNA damage (Figure 5). In a study conducted by Liao et al. (2019), the impact of glutamine-dependent anaplerosis on cell fate, specifically proliferation and senescence, was investigated using the pharmacological inhibitor amino-oxyacetate (AOA). Using WI38 normal human embryonic fibroblast cells, the researchers found that exposure to AOA led to mTORC1 inactivation and mTORC2 activation within the first day. This was followed by cell cycle arrest from day 2 to 6 and cellular senescence from day 4 to 6. Interestingly, these effects could be rescued by supplementing with anaplerotic factors such as αKG, pyruvate, or oxaloacetate, and the effects were independent of the ROS scavenger N-acetyl-cysteine (NAC). AOA-induced cellular senescence in WI38 cells was associated with increased protein levels of p53, p21^CIP1/Waf1^, and p16^INK4a^, accompanied by decreased levels of Rb protein. Supplementation with αKG could block these effects. Similar effects on cell proliferation and protein levels of P-Rb-S807/811 and Rb were observed when exposing p16^INK4a^-deficient U2OS human osteosarcoma cells and p16INK4A-knockdown WI38 cells to AOA. Interestingly, AOA failed to induce cellular senescence in U2OS cells, but it still manifested this effect in p16^INK4a^-knockdown WI38 cells, coinciding with the presence of p16 antibody-reactive p12. This study highlights the critical role of glutamine-dependent anaplerosis in cell growth and its association with mTORC1 and mTORC2 signaling. Furthermore, it demonstrates its involvement in cellular senescence, particularly in relation to p16^INK4a^ (178).

Jeong et al. (2013) conducted a study revealing that defects in mitochondrial glutamine metabolism lead to delayed cell cycle arrest and impaired DNA repair, resulting in the accumulation of DNA damage and the potential induction of permanent cell cycle arrest (179). Kim et al. (2020) emphasize the critical role of mitochondrial glutamine metabolism in DNA damage-induced senescence. The authors demonstrate that glutamine anaplerosis contributes to the induction of senescence and reveal that the regulation of mTOR activity by glutamine anaplerosis determines the extent of senescence induction. Importantly, their findings suggest that enhancing glutamine anaplerosis can promote cancer cells to undergo senescence following chemotherapy, offering a promising avenue for future therapeutic strategies. Targeting mitochondrial glutamine metabolism presents an opportunity to disable the proliferative capacity of cancer cells with reduced cytotoxicity and side effects associated with conventional treatments (180). These findings support the idea that elevating glutamine metabolism may synergize with DNA-damaging therapies, such as chemotherapy or radiation therapy, providing promising implications for cancer treatment. Furthermore, these results align with previous research by Jeong et al. (2013) highlighting the repression of mitochondrial glutamine metabolism as a key regulator of the DDR (179, 181), underscoring the significance of elevated glutamine anaplerosis through DMKG treatment in increasing senescence induction following DNA damage. It is crucial to note that high dosages of DNA damaging agents may induce cell death instead of senescence. Consequently, future research aims to investigate the involvement of mitochondrial glutamine metabolism in DNA damage-induced cell death and the contribution of glutamine anaplerosis to the decision between senescence and cell death.

Glutaminolysis is a metabolic pathway that converts glutamine into glutamate and ammonia through the action of the enzyme glutaminase 1 (GLS1) (182). The resulting glutamate is further transformed into αKG which enters the TCA cycle to generate ATP. This pathway is particularly significant in rapidly proliferating cells, including cancer cells, as it serves as an alternative energy source and supports cell growth and survival. Inhibiting glutaminolysis has shown promise in ameliorating aging-related characteristics and improving mitochondrial function in age-related diseases (183). A study by Johmura et al. (2021) investigated the effects of inhibiting glutaminolysis, mediated by GLS1, on senescent cells. The researchers discovered that senescent cells heavily rely on glutaminolysis for energy production and survival. By employing a pharmacological inhibitor to impede glutaminolysis, they observed selective cell death in senescent cells while sparing non-senescent cells. The study furnished evidence from *in vitro* and *in vivo* experiments, along with genetic mouse models, substantiating the effectiveness of inhibiting glutaminolysis in targeting senescent cells. Furthermore, the authors reported improvements in tissue function and the mitigation of age-related disorders such as frailty, osteoporosis, and cardiovascular dysfunction through senolysis achieved by inhibiting glutaminolysis. These findings propose that targeting glutaminolysis holds promise as a therapeutic strategy to selectively eliminate senescent cells and enhance health outcomes in various age-associated disorders (183, 184).

## 5. NAD^+^ metabolism in cellular senescence

### 5.1. NAD^+^ levels decline with aging

During glycolysis, NAD^+^ is reduced to its active form, NADH, which is then oxidized in the ETC to generate ATP. NADH is also involved in essential energy-related processes such as the TCA cycle and OXPHOS pathways (49–51). NAD^+^ serves as a crucial coenzyme for mitochondrial function, acting as an electron transporter and a substrate for important enzymes such as poly(ADP-ribose) polymerase (PARP) and sirtuins (SIRTs). NAD^+^ plays a vital role in DNA repair, metabolism, and longevity. With aging, there is a significant decline in NAD^+^ and NADH levels (185–190). This NAD^+^ depletion impairs DNA repair mechanisms, contributes to mitochondrial dysfunction, increased oxidative stress, and disrupted metabolism and nutrient sensing pathways (191–194). The depletion of NAD^+^ mediated by PARP and decreased activity of SIRT1 are associated with age-related increases in oxidative damage to nuclear DNA (139, 142–145). Studies have shown that supplementation with NAD^+^ precursors can extend lifespan in various species (140, 195–198).

Braidy et al. (2011) investigated the effects of aging on NAD^+^ metabolism, oxidative stress, and SIRT1 activity in female Wistar rats. They examined various organs (the heart, lung, liver, and kidney) and observed a significant decline in intracellular NAD^+^ levels and the NAD:NADH ratio during middle age (12 months) compared to young rats (3 months). This decline was accompanied by increased oxidative damage, impaired antioxidant capacity, DNA damage, reduced SIRT1 activity, elevated acetylated p53 levels, and impaired mitochondrial activity. The study emphasizes the importance of maintaining sufficient NAD^+^ levels for longevity and suggests a relationship between NAD^+^ depletion, oxidative stress, and SIRT1 activity during the aging process in Wistar rats (185).

In a subsequent study by Braidy et al. (2014), the impact of aging on intracellular NAD^+^ metabolism in different brain regions of female Wistar rats was examined. The study found a significant decline in intracellular NAD^+^ levels and NAD:NADH ratio within the central nervous system during aging. This decline was accompanied by increased lipid peroxidation, protein oxidation, reduced antioxidant capacity, elevated levels of H2AX hyperphosphorylation, increased expression of PARP1 and PARP2, enhanced CD38 activity, decreased ATP levels, impaired SIRT1 function, reduced mitochondrial complex activity, and impaired mitochondrial respiration rate across various brain regions. CD38 was identified as a key regulator of cellular NAD^+^ levels in rat neurons, suggesting its potential as a therapeutic target for age-related neurodegenerative diseases. These findings provide insights into NAD^+^ metabolism in the aging brains of Wistar rats and highlight potential targets to influence brain senescence, emphasizing the importance of understanding NAD^+^ metabolism for promoting brain health during the aging process (186). Of note, concerns have been raised by the PLOS ONE Editors (2022) regarding the validity and reliability of these findings (199).

In a study conducted by Massudi et al. (2012), the impact of aging on oxidative stress and NAD^+^ metabolism in human tissues was investigated. The study analyzed pelvic skin samples from individuals of different age groups and made several important findings. Both males and females exhibited a strong negative correlation between NAD^+^ levels and age, as well as a strong positive correlation between DNA damage and age, while lipid oxidation levels increased with age in males but not females. In males, PARP activity significantly increased with age and was inversely associated with tissue NAD^+^ levels, although these associations were less prominent in females. SIRT1 activity negatively correlated with age in males but not females. Interestingly, post-pubescent males showed positive correlations between lipid peroxidation and DNA damage, as well as between PARP activity and NAD^+^ levels. The study provides evidence supporting the hypothesis that the hyperactivation of PARP, due to the accumulation of oxidative DNA damage during aging, may contribute to increased NAD^+^ catabolism in human tissue. This NAD^+^ depletion potentially plays a significant role in the aging process by limiting energy production, DNA repair, and genomic signaling (189).

In a recent review by Peluso et al. (2021), the evidence regarding the decline of NAD^+^ levels with aging was critically examined. The authors raise concerns about the generalizability of the claim that NAD^+^ levels universally decrease with age, highlighting the limited and often tissue-specific nature of the supporting evidence. The review emphasizes the importance of conducting larger, preferably longitudinal, studies to comprehensively investigate NAD^+^ levels throughout the aging process in various tissues. The authors suggested that such studies are vital for advancing our understanding of NAD^+^ metabolism during aging and facilitating the development of more precise pharmacological interventions (200).

### 5.2. NAD^+^ depletion contributes to mitochondrial dysfunction

Mitochondrial dysfunction, a hallmark of aging, remains a debated topic when it comes to understanding its underlying causes. The theory of mitochondrial integration suggests that communication between the nucleus and mitochondria is vital for cellular metabolism, as they encode different subunits of the OXPHOS system. In a study by Gomes et al. (2014), researchers investigated the impact of NAD^+^ depletion on nuclear-mitochondrial communication during aging. The findings revealed that NAD^+^ depletion creates a pseudo-hypoxic state, disrupting this communication and negatively affecting cellular functions, thus contributing to the aging process. The study also observed a specific loss of mitochondrial-encoded OXPHOS subunits during aging, while nuclear-encoded subunits remained unaffected. This loss is attributed to an alternative pathway of nuclear-mitochondrial communication, independent of PGC-1α/β. Under normal conditions, this pathway is activated by nuclear NAD^+^ depletion and the accumulation of HIF-1α, resembling the Warburg effect. Deletion of SIRT1 accelerates this process, whereas increasing NAD^+^ levels in older mice restores mitochondrial function to that of young mice in a SIRT1-dependent manner. Overall, the study highlights the significance of the pseudohypoxic state in disrupting PGC-1α/β-independent nuclear-mitochondrial communication in the age-related decline of mitochondrial function. It emphasizes the critical role of maintaining optimal NAD^+^ levels in preserving efficient nuclear-mitochondrial communication and mitigating age-related effects (187).

Mitochondria play a crucial role in senescence-associated alterations, and mitochondrial dysfunction triggers a specific proinflammatory phenotype known as mitochondrial dysfunction-associated senescence (MiDAS), as described by Giuliani et al. (2017). Their research demonstrated that mitochondrial dysfunction promotes ROS production, DNA damage, and inflammation, contributing to cellular senescence. Importantly, they found that inhibiting mitochondrial ROS production with a mitochondrial-targeted antioxidant can prevent cellular senescence, suggesting that targeting mitochondrial dysfunction holds promise for preventing age-related diseases. It is noteworthy that MiDAS differs from the SASP due to the absence of an IL-1/NF-κB-dependent mechanism. MiDAS is also believed to contribute to the aging process itself, as the accumulation of dysfunctional mitochondria in aging cells is thought to drive the SASP. In MiDAS, a reduced NAD^+^/NADH ratio is believed to activate adenosine monophosphate-activated protein kinase (AMPK) and p53 (201–203).

Qian et al. (2019) investigated the effects of mitochondrial dysfunction-induced ROS on the nucleus using a chemoptogenetic approach. By inducing rapid mitochondrial dysfunction through light stimulation, the researchers observed respiratory loss, decreased ETC activity, and mitochondrial fragmentation. Interestingly, they discovered a persistent secondary wave of mitochondrial superoxide and hydrogen peroxide production that lasted for more than 48 h after initial singlet oxygen exposure. Ratiometric analysis indicated the presence of hydrogen peroxide in the nucleus, suggesting its diffusion from dysfunctional mitochondria. Although mitochondrial DNA damage and nuclear oxidative stress were observed, no significant nuclear DNA strand breaks or apoptosis were detected. However, targeted analysis revealed fragile telomeres, telomere loss, and the presence of telomere dysfunction-induced foci (TIFs) positive for the DNA repair protein 53BP1. These findings suggest that DNA double-strand breaks specifically occurred in telomeres because of mitochondrial dysfunction. Furthermore, the study showed that ATM-mediated DDR signaling was activated in response to these telomere defects. Interestingly, inhibiting ATM worsened mitochondrial dysfunction and increased cell sensitivity to apoptotic cell death induced by the chemoptogenetic approach. These results highlight the sensitivity of telomeres to hydrogen peroxide generated by dysregulated mitochondria and reveal an important mechanism of telomere-mitochondria communication involved in the pathophysiology of human diseases associated with mtROS (204).

PARP1 is an important sensor of DNA damage that rapidly responds to such damage by initiating various cellular processes, including DNA damage repair and chromatin organization. In a study by Murata et al. (2019), advanced fluorescence imaging and laser micro irradiation techniques were employed to investigate the effects of PARP activation. The researchers observed a swift increase in the fraction of bound NADH throughout the cell following nuclear DNA damage, which resulted from the depletion of NAD^+^ mediated by PARP. This metabolic shift was associated with a transition from glycolysis to OXPHOS. Importantly, inhibiting OXPHOS, but not glycolysis, triggered a process called parthanatos, characterized by rapid ATP depletion caused by PARP activation. These findings reveal a novel prosurvival response to PARP activation involving alterations in cellular metabolism (205, 206). It should be note that PARP activation is also implicated in mitochondrial dysfunction (207).

In a study by Kotla et al. (2021), the impact of mtROS-induced telomere DNA damage following chemo-radiation on nucleus-mitochondria communication was investigated. The researchers found that the p90RSK-ERK5 S496-NRF2 pathway is involved in this process. They demonstrated the critical role of p90RSK-mediated ERK5 S496 phosphorylation and subsequent reduction of NRF2 transcriptional activity in PARP activation, mitochondrial stunning, and mitochondrial ROS production through the establishment of a positive feedback loop between the nucleus and mitochondria. Activation of this nuclear-mitochondria feedback loop reprograms monocytes toward a SASP, characterized by increased telomere DNA damage and heightened sensitivity to ROS. The study also showed that inhibiting PARP effectively suppressed radiation-induced mitochondrial stunning, monocyte priming, and CAD. Based on these findings, PARP inhibitors emerge as promising candidates not only as radio-sensitizers but also as effective agents to mitigate cardiovascular events following radiation therapy (131).

In a study conducted by Fang et al. (2014), the researchers investigated defective mitophagy in Xeroderma pigmentosum group A (XPA), a genetic disorder associated with impaired DNA repair. The study revealed that XPA is characterized by increased PARP1 activity, leading to excessive consumption of NAD^+^ and subsequent reduction in SIRT1 levels. These changes disrupt the process of mitophagy, which is responsible for eliminating damaged mitochondria. The findings highlight the significance of PARP1, NAD^+^, and SIRT1 in maintaining proper mitophagy and suggest their dysregulation as contributing factors to mitochondrial dysfunction and the development of XPA. Understanding the molecular mechanisms involved in defective mitophagy holds potential for developing therapeutic strategies not only for XPA but also for related disorders characterized by mitochondrial dysfunction, neurodegeneration, and aging (188).

In another study conducted by Fang et al. (2016), the researchers investigated the implications of increased PARylation, low NAD^+^ levels, and mitochondrial dysfunction in Ataxia telangiectasia (A-T) using mice and worms as model organisms. The study revealed that interventions aimed at restoring intracellular NAD^+^ levels had beneficial effects on A-T neuropathology. These treatments improved neuromuscular function, delayed memory loss, and extended lifespan in both animal models. Furthermore, the study demonstrated that elevated intracellular NAD^+^ levels stimulated neuronal DNA repair mechanisms and enhanced mitochondrial quality through mitophagy. By establishing a connection between the accumulation of DNA damage and mitochondrial dysfunction, the research provided insights into the intricate interplay of nuclear DNA damage-induced nuclear-mitochondrial signaling. This signaling was identified as a critical factor contributing to the premature aging observed in A-T. The finding not only deepened our understanding of A-T's pathophysiology but also proposed potential therapeutic interventions by targeting NAD^+^ replenishment and promoting mitochondrial quality control. This work established valuable connections between fundamental aging processes and the specific molecular mechanisms associated with A-T, laying the foundation for further investigations and the development of targeted treatments (190).

### 5.3. The role of CD38 in NAD^+^ depletion during aging

CD38, a highly active ectoenzyme involved in NAD^+^ metabolism, has emerged as a key player in the age-related NAD^+^ depletion. In a study conducted by Chini et al. (2019), the relationship between cellular senescence, CD38 expression, and NAD^+^ depletion during aging was investigated. Surprisingly, the researchers found that senescent cells themselves did not express high levels of CD38. However, factors secreted by these cells, including SASP factors, induced the expression of CD38 mRNA and protein in non-senescent cells such as ECs and bone marrow-derived macrophages. This induction led to increased CD38-NADase activity in these cells. These findings suggest a potential association between cellular senescence and the age-related NAD^+^ depletion. The study provides valuable insights into the underlying mechanisms of NAD^+^ depletion during aging and highlights the significance of CD38 in this process. Furthermore, this research paves the way for future investigations and interventions aimed at counteracting age-related NAD^+^ depletion (208–210). Notably, CD38 inhibitors have shown promise in rescuing NAD^+^ depletion and improving metabolic outcomes in mice (211). Additionally, the delivery of the extracellular isoform of nicotinamide phosphoribosyltransferase (NAMPT), a rate-limiting enzyme in the NAD^+^ salvage pathway, through EVs, has demonstrated beneficial effects on mouse lifespan (212). EVs, membrane-coated nanoparticles released by various cell types including ECs, have the ability to transport and deliver functional proteins and nucleic acids in a paracrine and systemic manner (213).

### 5.4. NAD^+^ metabolism induces the SASP

In senescent HDFs, the regeneration of NAD^+^ leads to the upregulation of LDH, facilitating the release of lactate. This lactate release plays a crucial role in preventing glycolysis from stalling due to lactate accumulation and intracellular acidification. These findings suggest that supplementation with malate, an intermediate of the TCA cycle, may delay senescence and potentially extend lifespan. Similar effects have been observed in C. elegans and D. melanogaster when supplemented with aKG and oxaloacetate, other TCA cycle intermediates. However, further research is required to determine the translation of these findings to mammals (214, 215).

Nacarelli et al. (2019) conducted a study demonstrating the direct influence of intracellular NAD^+^ levels on the SASP in cells undergoing oncogene-induced senescence (OIS). They observed that chromatin remodeling during OIS increases chromatin accessibility and activates the chromatin-binding protein HMGA1. HMGA1, in turn, upregulates the expression of NAMPT, a key enzyme involved in NAD^+^ metabolism. Inhibition of NAMPT leads to reduced glycolysis, mitochondrial respiration, and a decrease in the NAD^+^/NADH ratio in OIS cells. The decrease in the NAD^+^/NADH ratio activates AMPK, which inhibits p38MAPK, a regulator of the SASP. Therefore, a high NAD^+^/NADH ratio in OIS promotes the activation of SASP. Interestingly, different models of senescence show contrasting observations, suggesting that NAD^+^ metabolism specifically controls distinct subsets of SASP factors. These findings highlight the critical role of NAD^+^ metabolism in regulating the SASP and suggest that targeting NAD^+^ metabolism may hold promise for treating age-related diseases (194).

Lautrup et al. (2019) conducted a comprehensive review exploring the significance of NAD^+^ in cellular metabolism and ATP production. The authors focused on the impact of NAD^+^ depletion on brain aging, age-related cognitive decline, and neurodegenerative diseases such as Alzheimer's disease, Parkinson's disease, and Huntington's disease. The authors examined the diverse functions of NAD^+^ in DNA repair, gene expression regulation, maintenance of mitochondrial function, and modulation of neuronal signaling pathways. The review emphasized how NAD^+^ depletion disrupts cellular metabolism, increases oxidative stress, triggers neuroinflammation, and contributes to neuronal dysfunction, thereby promoting the progression of neurodegenerative disorders. The authors discussed various strategies to enhance NAD^+^ levels, including the utilization of NAD^+^ precursors such as nicotinamide riboside (NR) and nicotinamide mononucleotide (NMN). They also explored the potential of targeting NAD^+^-consuming enzymes such as SIRTs and PARPs for therapeutic intervention. Their comprehensive review underscores the critical role of NAD^+^ in cellular metabolism and its implications for brain aging and neurodegenerative diseases. The strategies for increasing NAD^+^ levels and targeting NAD^+^-related enzymes offer promising therapeutic avenues in the field of neurodegenerative disorders (216).

## 6. Metabolic regulation of senescence-induced tumor suppression

Cellular senescence triggered by various oncogenic insults, such as the activation of the ras proto-oncogene, serves as a crucial mechanism to prevent tumorigenesis (217–220). Moreover, alterations in cellular carbohydrate metabolism, including the phenomenon of aerobic glycolysis, have been associated with oncogenic transformation. In an *in vitro* model of senescence using human fibroblasts, Zwerschke et al. (2003) demonstrated that senescent cells undergo a metabolic imbalance characterized by a significant reduction in levels of ribonucleotide triphosphates, including ATP. These molecules are essential for nucleotide biosynthesis and cellular proliferation. These findings underscore the critical role of metabolic changes in senescence-induced tumor suppression (156).

### 6.1. The complexity and context dependency of metabolic regulation of oncogene-induced senescence (OIS)

OIS is characterized by permanent cell cycle arrest and a pro-inflammatory phenotype, playing a crucial role in tumor suppression. The metabolic behavior of OIS cells is significantly influenced by key senescence effectors, including p53 and pRb. By understanding the metabolic changes occurring in OIS cells, we can gain valuable insights into the underlying mechanisms of tumor suppression and develop targeted therapeutic strategies to prevent cancer development. These insights are essential for unraveling the intricate relationship between metabolism and senescence-mediated tumor suppression.

In a study by Quijano et al. (2012), Ras-induced senescent human fibroblasts (OIS) exhibited significant alterations in intracellular long-chain fatty acids, indicating a distinct metabolic characteristic. Despite an increase in free fatty acids (FAAs), *de novo* fatty acid synthesis decreased in OIS cells. Assessment of FAO confirmed elevated mitochondrial oxygen consumption, particularly in FAO, supporting previous findings linking specific long-chain fatty acids to mitochondrial oxidation and energy production (221, 222). OIS cells displayed higher mitochondrial oxygen consumption, primarily in FAO. The shift towards FAO was accompanied by increased activity of CPT1, the outer mitochondrial membrane protein that catalyzes the rate-limiting step in FAO. Inhibiting CPT1 activity restored the metabolic rate to a pre-senescent state and specifically suppressed the pro-inflammatory state associated with OIS, including the development of SASP and preventing senescence in these cells. These findings indicate significant metabolic and bioenergetic changes, particularly in fatty acid metabolism, in Ras-induced OIS cells, contributing to the observed inflammatory phenotype (223). Recent research suggests that changes in FAA levels and oxidation may influence tumor aggressiveness (224, 225). Additionally, FAAs and certain phospholipid-derived molecules may play a role in determining the longevity of simple organisms, bridging the connection between senescence and metabolism. Inhibiting the increase in mitochondrial FAO associated with senescence selectively suppresses the secretory state linked to OIS. Elevated cytokine production was observed in IMR-90 cells undergoing Ras-mediated senescence, while cells undergoing RS did not exhibit the same effect, consistent with previous findings in this cell line. Further investigations are needed to determine the generalizability of the relationship between FAO and senescence-associated inflammation. Ras-induced senescent cells showed increased cytokine production, with IL-1β secretion remaining unaffected by genetic or pharmacological inhibition of CPT1. IL-1β secretion involves the inflammasome, and mitochondrial ROS may be implicated (226–228). These findings suggest a broader role for mitochondrial activity in maintaining the inflammatory state than previously assumed (223).

In a study conducted by Kaplon et al. (2013), the researchers investigated the role of pyruvate dehydrogenase (PDH) in OIS triggered by the BRAFV600E oncogene, commonly found in melanoma and other cancers. They observed that in BRAFV600E-induced OIS cells, the inhibitory enzyme of PDH, pyruvate dehydrogenase kinase 1 (PDK1), was suppressed, while the activating enzyme pyruvate dehydrogenase phosphatase 2 (PDP2) was induced. This led to the activation of PDH, resulting in increased utilization of pyruvate in the TCA cycle, enhanced respiration, and elevated redox stress. The reversal of these processes coincided with the abrogation of OIS, representing a critical step towards preventing oncogenic transformation. Furthermore, normalizing the expression levels of either PDK1 or PDP2 suppressed PDH activity and eliminated OIS, providing a means to induce OIS in BRAFV600E-expressing cells. Depleting PDK1 also led to the regression of established melanomas and eliminated melanoma subpopulations that were resistant to targeted BRAF inhibition. These findings revealed a mechanistic link between OIS and a crucial metabolic signaling pathway, offering potential avenues for intervention and treatment of BRAFV600E-induced OIS (229).

In their study, Takebayashi et al. (2015) explored the role of retinoblastoma protein (RB) in promoting metabolic flow through glycolysis and mitochondrial OXPHOS in OIS cells. Through real-time metabolic monitoring, metabolome analysis, and gene expression profiling, the researchers observed an enhanced metabolic flow in OIS-induced fibroblasts. The study uncovered RB as a key regulator in upregulating glycolytic genes in OIS cells. Depletion of RB led to the downregulation of several glycolytic genes and a reduction in metabolites derived from the glycolytic pathway. Remarkably, when RB or downstream glycolytic enzymes were depleted, both mitochondrial OXPHOS and glycolytic activities were abolished in OIS cells. These findings highlight the significant role of RB in metabolic remodeling and the maintenance of active energy production in OIS cells. They contribute to our understanding of the metabolic alterations associated with OIS and provide valuable insights into the mechanisms underlying cellular senescence (230).

By classifying senescence forms based on their triggers, metabolic studies have uncovered diverse metabolic behaviors of different types of OIS cells. In some cases, there is a metabolic shift towards the TCA cycle and OXPHOS as the primary pathways for glucose metabolism (229, 230). These metabolic alterations contribute to the tumor-suppressive phenotype of OIS cells, which differs from the metabolic behavior of tumor cells prioritizing lactate production or the PPP to support rapid proliferation through nucleotide synthesis (223, 231). However, in other cases, OIS cells exhibit reduced OXPHOS and a shift towards glycolysis, along with elevated rates of glucose consumption, lactate production, and oxygen consumption. These cells also show increased expression levels of glycolytic and OXPHOS enzymes, higher levels of ROS, and an increase in mitochondrial mass. These metabolic changes resemble the metabolic profile observed in cancer cells and may contribute to the resistance to cell cycle arrest, as well as the ability of OIS cells to promote tumor growth through the induction of SASP and the secretion of growth factors and cytokines (223). These contrasting observations suggest that OIS cells can exhibit a metabolic profile like that observed in cancer cells (232). These discrepancies highlight the complexity of metabolic alterations in OIS cells, which can be context-dependent and influenced by factors such as the specific oncogene or stressor that triggered senescence, as well as the cellular context and microenvironment. Understanding the diverse metabolic profiles of OIS cells is essential for unraveling the underlying mechanisms and developing targeted therapeutic strategies for cancer and age-related diseases. Further research is needed to fully understand the metabolic changes associated with OIS and how they evolve when cells overcome the cell cycle arrest associated with OIS (231).

### 6.2. Metabolic regulation of therapy-induced senescence (TIS): implications for treatment of age-related disorders

TIS is a state of stable cell cycle arrest that occurs in viable cells following cancer treatments such as chemotherapy and radiation therapy. While TIS can have long-term benefits, the harmful properties of senescent cancer cells make their elimination a therapeutic priority. In a study conducted by Demaria et al. (2017) using a mouse lymphoma model, the metabolic changes associated with TIS were investigated. The researchers found that TIS-competent lymphomas, but not TIS-incompetent lymphomas lacking the H3K9 histone methyltransferase Suv39h1, exhibited increased glucose utilization and higher ATP production after senescence-inducing chemotherapy. This increase in energy production was linked to proteotoxic stress caused by the SASP. TIS cells expressing SASP induced endoplasmic reticulum stress and unfolded protein response (UPR), leading to enhanced autophagy-mediated clearance of toxic proteins, which required significant energy. The study demonstrated that blocking glucose utilization or autophagy selectively eliminated TIS lymphomas by inducing endoplasmic reticulum-related apoptosis mediated by caspase-12 and caspase-3. These findings suggest that targeting the metabolic demands associated with TIS induction may lead to tumor regression and improved treatment outcomes *in vivo*. They highlight the potential of exploiting metabolic vulnerabilities as a therapeutic strategy for eliminating senescent cancer cells and enhancing treatment efficacy. Recent studies have shown promise in targeting cancer cell metabolic vulnerabilities during TIS, revealing synthetic lethality and providing further support for this approach (162, 233, 234).

## 7. Metabolic regulation of EC senescence

### 7.1. The role of EC senescence in CVD

Age-related EC dysfunction is characterized by a shift towards a vasoconstrictive, proinflammatory, and prothrombotic environment. It has been widely recognized as a significant precursor to the development of CVD (235–239), leading to impaired regulation of vascular homeostasis. EC dysfunction is predominantly attributed to EC senescence, which has been implicated in the pathogenesis of various age-related CVD, including stroke, vascular dementia, macular degeneration, obstructive sleep apnea, atherosclerosis, myocardial infarction, pulmonary hypertension, hypertension, diabetes, renal failure, peripheral arterial disease, and diabetic complications (193, 240–255). The presence of EC senescence has been detected in human tissues across different pathophysiological conditions (256). For instance, Minamino et al. (2002) reported the presence of EC senescence in human atherosclerotic plaques of the aorta and coronary arteries (247), while Villaret et al. (2010) observed EC senescence in adipose tissues of obese individuals (257). Studies by Hwang et al. (2019) and Kopacz et al. (2020) demonstrated that senescent ECs exhibit structural and functional changes, including increased protein aggregation, compared to young cells (258, 259). Senescent ECs also display pro-inflammatory, pro-thrombotic, vasoconstrictive phenotypes, contributing to the promotion of age-related diseases (260). EC senescence has also been observed in animal models, such as in single and double balloon denudation of rabbit carotid arteries (261) and Zucker diabetic rats (262). In the study by Brodsky et al. (2004), a sixfold increase in the number of senescent ECs was observed in Zucker diabetic rats at 22 weeks of age, accompanied by the induction of p53, p21, and p16 (262). Using single-cell RNA sequencing, Kiss et al. (2020) found that approximately 10% of cerebral-microvascular ECs undergo cellular senescence in the brain of 28-month-old mice (263). The biological and clinical significance of EC senescence underscores the importance of developing strategies to identify and therapeutically target senescent ECs. The mechanisms underlying EC senescence involved multiple layers, including chromatin, DNA, RNA, and protein levels (264, 265). Various factors contribute to EC senescence, including disturbed flow patterns, cancer therapies, inflammaging, NAD^+^ depletion, and others (91, 264).

### 7.2. EC metabolism in angiogenesis and vascular disorders

Angiogenesis, the process of generating new blood vessels from pre-existing ones, is a vital mechanism in both normal physiological processes and pathological conditions (266). Traditionally, angiogenesis has been primarily associated with the coordinated migration and proliferation of ECs, which are stimulated by the activation of vascular endothelial growth factor (VEGF). This process is crucial for wound healing, normal functioning of the female reproductive system, but can also contribute to conditions such as cancer and chronic inflammation. During angiogenesis, specific ECs undergo phenotypic changes and differentiate into two distinct cell types: tip cells and stalk cells. Tip cells guide the elongation and branching of developing vessels by directing the behavior of stalk cells (267–269). Given the energy-intensive nature of angiogenesis, ECs undergo significant metabolic adaptations as they transition from a quiescent state to support the increased energy and biomass requirements during vessel branching (270). Recent research has shed light on the critical role of metabolic regulation in ECs, uncovering key metabolic pathways and regulatory mechanisms that drive angiogenesis (271–275).

ECs exhibit phenotypic specialization to fulfill various functions and give rise to other cell types that are essential for blood and heart development. The specific characteristics of their vascular regions have been extensively reviewed by Dejana et al. (2017) and play a significant role in these processes (276). To gain a comprehensive understanding of the global specialization of ECs, Chi et al. conducted a study using DNA microarrays to analyze the expression profiles of 53 cultured ECs. They identified distinct and characteristic gene expression profiles in ECs derived from different blood vessels and microvascular ECs from various tissues. Importantly, they found significant differences in gene expression patterns that distinguished ECs in large vessels from microvascular ECs. The researchers discovered specific gene clusters that were selectively expressed in arterial and venous endothelium, with Hey2 being particularly expressed in arterial ECs and influencing the expression of several arterial-specific genes. Furthermore, they observed preferential expression of genes involved in establishing left/right asymmetry in venous ECs, suggesting a coordination between vascular differentiation and the development of body plans. Tissue-specific expression patterns in microvascular ECs from different tissues indicated the presence of distinct differentiated cell types, contributing to the localized physiology of their respective organs and tissues. In the microvasculature, ECs play a crucial role in facilitating the exchange of gases and macromolecules between the circulation and tissues. Additionally, they modify circulating mediators such as lipoproteins and angiotensin, further highlighting their functional diversity and importance in maintaining tissue homeostasis (277).

ECs possess unique metabolic characteristics, exhibiting lower mitochondrial mass compared to other cell types and a preference for glycolysis as their primary ATP generation pathway, even in the presence of sufficient oxygen (278, 279). The role of EC metabolism in driving angiogenesis, in addition to well-established angiogenic growth factors like VEGF, has been established. VEGF signaling promotes glycolysis in ECs, resulting in increased expression of glucose transporter GLUT-1 and the synthesis of fructose-2,6-bisphosphate (F2,6BP) mediated by the enzyme PFKFB3, a critical regulator of glycolysis (280, 281). Glycolysis provides the necessary energy for the competitive behavior of ECs at the leading edge of growing vessel sprouts, while FAO controls nucleotide synthesis and EC proliferation in the trailing stalk of the sprout. The rate of glycolysis in ECs is influenced by their specialization and proliferation rate. Beyond ATP generation, glycolysis also plays a vital role in modulating EC function and phenotype. The upregulation of the glycolytic pathway by HIF-1α, coupled with the downregulation of PDH, leads to lactate accumulation, stabilizes HIF-1α, and exerts a paracrine proangiogenic effect on neighboring ECs. Disruptions in EC metabolism contribute to vascular disorders, including EC dysfunction and excessive angiogenesis. However, our current understanding of EC mitochondrial metabolism in this context is limited. While evidence supports impaired angiogenesis under glucose-deprived conditions (282–285), recent studies have demonstrated that inhibiting the ETC reduces EC proliferation. Interestingly, this effect is associated with a decline in the NAD^+^/NADH ratio rather than a decrease in ATP availability (286, 287).

Manipulating glycolysis-related enzymes or regulators can have a significant impact on the phenotype of ECs. For instance, FOXO1 suppresses c-myc signaling, leading to reduced glycolysis and the maintenance of ECs in a quiescent state. Disrupting the FGFR/c-myc axis downregulates hexokinase 2 (HK2), impairing EC proliferation and migration. The balance between the two isoforms of PK is crucial for determining the fate of pyruvate. Increased expression of PKM2 promotes aerobic glycolysis, inducing a more proliferative state in ECs. Under physiological conditions, laminar blood flow enhances NO-mediated S-nitrosylation of PKM2, resulting in reduced glycolysis and increased substrate flux through the PPP. In low-replication-rate cells like quiescent ECs, balanced PPP activity promotes antioxidant responses by generating NADPH as reducing equivalents. Further studies are needed to unravel the intricate interplay between EC metabolism, mitochondrial function, and angiogenesis, which may provide new insights for developing therapeutic strategies for vascular disorders (288).

### 7.3. FAO and redox homeostasis as metabolic characteristics of quiescent ECs

ECs possess the necessary proteins for efficient fatty acid uptake and intracellular transport. In the microcirculation, ECs utilize lipoprotein lipase to extract fatty acids from circulating lipoproteins. Interestingly, proliferating ECs prioritize FAO for *de novo* dNTP synthesis instead of OXPHOS (278). However, in quiescent ECs, FAO is upregulated to maintain redox homeostasis and support the TCA cycle through NADPH regeneration through the activity of the malic enzyme, rather than for energy or biomass production. Kalucka et al. (2018) conducted a study that revealed the critical role of FAO in quiescent ECs. They demonstrated that impaired FAO control in CPT1AΔEC mice led to EC dysfunction, increased oxidative stress, and heightened susceptibility to inflammatory diseases. The authors identified Notch1 as a key regulator of FAO for redox balance in quiescent ECs. Notably, supplementation of acetate restored EC quiescence and mitigated oxidative stress-induced dysfunction in CPT1AΔEC mice, suggesting potential therapeutic approaches. This research provides insights into the protective function of FAO in quiescent ECs against oxidative stress and its implications for vascular disorders (289).

### 7.4. Glutamine metabolism in ECs

Our understanding of EC metabolism has made significant progress, particularly in relation to glycolysis and FAO. However, there is still limited knowledge regarding other metabolic pathways involved in angiogenesis. One such pathway that shows promise as a target for anti-angiogenic strategies focused on EC metabolism is glutamine metabolism. GLS1, an enzyme predominantly found in the mitochondria of ECs, plays a vital role in their proliferation through participation in glutamine metabolism. In a study by Huang et al. (2017), the researchers investigate the roles of glutamine and asparagine metabolism in EC function and vessel formation. Their findings demonstrate that depriving ECs of glutamine or inhibiting GLS1 leads to defects in vessel sprouting, including impaired proliferation, migration, and reduced ocular angiogenesis. Notably, the inhibition of glutamine metabolism does not cause energy depletion in ECs but instead affects the TCA cycle, macromolecule production, and redox homeostasis. However, replenishing the TCA cycle and supplementing with asparagine successfully restores the metabolic abnormalities and proliferation defects induced by glutamine deprivation. The study highlights the importance of glutamine metabolism in ECs as a nitrogen source for asparagine synthesis, which is essential for maintaining cellular homeostasis. Glutamine serves as a critical energy source and participates in various crucial processes within ECs. It generates αKG through deamination and transamination, which enters the TCA cycle for ATP generation, and contributes to nucleotide biosynthesis and the production of the antioxidant peptide glutathione. While ECs are capable of uptaking asparagine, silencing the enzyme asparagine synthetase (ASNS), responsible for converting glutamine-derived nitrogen and aspartate into asparagine, impairs EC sprouting even in the presence of glutamine and asparagine. Asparagine plays a critical role in restoring protein synthesis, suppressing endoplasmic reticulum stress, and reactivating mTOR signaling in glutamine-deprived ECs. These findings establish a novel connection between glutamine and asparagine metabolism in ECs during the process of vessel sprouting (290).

Glutamine supplementation has shown positive effects in ECs, including enhanced endothelial nitric oxide synthase (eNOS) activity, reduced inflammation, promotion of endothelial progenitor cell mobilization, and facilitation of vascular endothelium repair in diabetes-related ischemic injury (291). Restoring glutamine-dependent anaplerosis through GLS1 overexpression has demonstrated benefits in mitigating age-related bone loss and delaying EC senescence. Targeting GLS1 holds promise as a potential therapeutic approach for diseases characterized by abnormal EC proliferation.

Kim et al. (2017) conducted a study to investigate the role of glutamine in EC metabolism using genetic modifications and 13C tracing techniques. Their findings demonstrate that mice with EC-specific GLS1 deletion exhibited reduced angiogenesis. Deprivation of glutamine or inhibition of GLS1 in ECs effectively suppressed their proliferation while leaving cell migration unaffected, as migration relies on aerobic glycolysis rather than glutamine metabolism and GLS1 activity. This inhibitory effect was observed in ECs derived from various sources, such as the human umbilical vein, human aorta, and human microvasculature. Notably, glutamine deprivation or GLS1 inhibition resulted in a significant decrease in TCA cycle intermediates in ECs, without compensation from glucose-derived anaplerosis. Restoring TCA cycle intermediates by adding exogenous αKG rescued EC growth. The study also highlighted the importance of Rac1-dependent macropinocytosis, a process that enables cells to engulf fluid and nutrients, in obtaining NEAAs like asparagine. These findings contribute valuable insights into the intricate interplay between glucose and glutamine in EC metabolism, elucidating their distinct roles in this process (292).

In their study, Peyton et al. (2018) demonstrated that the absence of glutamine in the culture media or the inhibition of GLS1 activity or expression impeded the proliferation and migration of ECs derived from various sources, including the human umbilical vein, human aorta, and human microvasculature. GLS1 inhibition induced cell cycle arrest in the G0/G1 phase and caused a significant decrease in cyclin A expression. Restoring cyclin A expression through adenoviral-mediated gene transfer rescued the proliferative response of GLS1-inhibited ECs, while migration remained unaffected. This suggests that targeting cyclin A may serve as a potential therapeutic approach to enhance the proliferation of ECs when GLS1 activity is compromised. Glutamine deprivation or GLS1 inhibition increased ROS production and reduced the expression of heme oxygenase-1 (HO-1), an enzyme with cytoprotective properties. Moreover, GLS1 inhibition sensitized ECs to the cytotoxic effects of hydrogen peroxide, which could be prevented by overexpressing HO-1. Overall, the study highlights the critical role of GLS1 in promoting the proliferation, migration, and survival of human ECs irrespective of their vascular source. Cyclin A contributes to the proliferative action of GLS1, while HO-1 mediates its pro-survival effect. These findings suggest that GLS1 could be a promising therapeutic target for diseases characterized by abnormal EC proliferation, migration, and viability (293).

### 7.5. Metabolic regulation of EC senescence

#### 7.5.1. Oxidative glucose metabolism in vascular EC senescence

Oxidative glucose metabolism, also known as aerobic glycolysis or the TCA cycle, plays a significant role in cellular energy production when oxygen is available. The process begins with glycolysis in the cytoplasm, where glucose is converted into pyruvate. Subsequently, pyruvate enters the mitochondria and undergoes further processing in the TCA cycle. Within the TCA cycle, pyruvate is fully oxidized, leading to the generation of high-energy electron carriers, such as nicotinamide adenine dinucleotide (NADH) and flavin adenine dinucleotide (FADH2). These electron carriers produced in the TCA cycle are then utilized in the ETC, located in the IMM. The ETC utilizes these electron carriers to generate ATP through OXPHOS. As the electrons move through the ETC, they establish an electrochemical gradient, which drives the synthesis of ATP via the action of ATP synthase. Oxidative glucose metabolism is an efficient process that yields a significant amount of ATP. It serves as the primary energy-generating pathway in cells under aerobic conditions and plays a critical role in various cellular functions, including muscle contraction, brain function, and other energy-demanding processes. However, under conditions of limited oxygen availability or increased energy demand, pyruvate can be converted into lactate through the action of the enzyme LDH. This process, known as lactate fermentation or anaerobic glycolysis, regenerates the necessary cofactor NAD^+^ for glycolysis to continue. Lactate production is commonly observed in tissues with high glycolytic activity, such as muscles during intense exercise or rapidly dividing cancer cells (294).

Through the utilization of metabolic profiling, glucose flux measurements, and examination of key metabolic enzymes, Stabenow et al. (2022) conducted a study to investigate the metabolic characteristics of senescent ECs in comparison to young ECs. Surprisingly, their findings revealed an upregulation of both glycolytic and oxidative glucose metabolism in senescent ECs. Senescent ECs exhibited heightened glycolytic activity and lactate production, accompanied by an increased expression of LDHA. Moreover, there was an elevation in TCA cycle activity and mitochondrial respiration in senescent ECs. This increase in TCA cycle activity and mitochondrial respiration could be attributed to the reduced expression of PDHKs in senescent ECs, potentially resulting in augmented activity of the pyruvate dehydrogenase complex. Despite indications of mitochondrial dysfunction, such as elevated ROS production and increased mitochondrial mass, both cellular and mitochondrial ATP production were elevated in senescent ECs. Interestingly, when young ECs were induced to transition from glycolytic to oxidative glucose metabolism through pharmacological inhibition of PDHKs, premature senescence occurred. These findings suggest that alterations in cellular glucose metabolism may serve as a driver for senescence in ECs (295).

The mechanisms underlying the increased glycolytic activity in senescent ECs are not yet fully understood. However, it has often been associated with an elevated demand for macromolecule building blocks necessary for senescence-associated events, such as the SASP, cell enlargement, increased oxidative and ER stress. The upregulation of glycolysis may serve as a compensatory mechanism to overcome the reduced ATP production caused by mitochondrial dysfunction (24, 229, 296). Interestingly, although glycolysis was enhanced in senescent ECs, Stabenow et al. (2022) did not observe a concurrent increase in glucose metabolization via the PPP, which branches off after the initial step of glycolysis (Figure 1). The PPP is responsible for generating NADPH, a crucial player in redox homeostasis, and ribose 5-phosphate, a precursor for numerous biomolecules, including DNA and RNA. Consequently, the heightened glucose consumption in senescent ECs may not be directly linked to redox regulation or nucleotide synthesis (295).

#### 7.5.2. Fatty acid metabolism in EC senescence

Lin et al. (2022) conducted a study to investigate the role of fatty acid metabolism in EC senescence. Using replicative and H_2_O_2_-induced senescence models in human umbilical vein ECs (HUVECs), they observed suppressed FAO and disrupted fatty acid profiles, along with reduced expression of proteins involved in fatty acid uptake and mitochondrial entry, including CPT1A. Impairment of fatty acid metabolism by silencing CPT1A or using the CPT1A inhibitor etomoxir promoted EC senescence, as evidenced by increased levels of p53, p21, senescence-associated β-galactosidase, and decreased proliferating cells. Conversely, restoration of FAO through CPT1A overexpression or supplementation of short-chain fatty acids (SCFAs) acetate and propionate mitigated EC senescence. The study also identified acetyl-coenzyme A (acetyl-CoA) as a mediator of fatty acid metabolism in EC senescence. Inhibition of acetyl-CoA production accelerated H_2_O_2_-induced EC senescence, while supplementation of acetyl-CoA prevented it. Deficiency in acetyl-CoA resulted in alterations in acetylated protein profiles associated with cell metabolism and the cell cycle. Furthermore, *in vivo* treatment with acetate for four weeks demonstrated reduced blood pressure and alleviation of senescence-related phenotypes in the aortas of mice infused with Ang II. These findings indicate that fatty acid metabolism, including FAO and acetyl-CoA production, plays a crucial role in the regulation of EC senescence. Enhancing fatty acid metabolism may hold therapeutic potential for mitigating CVD associated with EC senescence (297).

#### 7.5.3. Glutaminolysis in EC senescence

Glutaminolysis is a vital metabolic pathway that enables the catabolism of glutamine to produce energy and metabolic intermediates within cells. This pathway assumes particular significance when glucose availability is limited or during cellular processes that require high energy demands. Glutaminolysis occurs in diverse cell types and involves the enzymatic conversion of glutamine to glutamate by the enzyme glutaminase. Subsequently, glutamate is further metabolized to generate energy and biosynthetic precursors essential for cellular functions (182).

Glutaminolysis has been found to significantly contribute to energy regeneration in senescent HUVECs (298). Senescent HUVECs exhibit increased rates of glutamine consumption, leading to elevated production of glutamate and lactate. However, these metabolic changes alone are insufficient to maintain ATP levels in these cells. Intriguingly, inhibiting glutaminase, the initial enzyme in the glutaminolytic pathway, induces cellular senescence even in early passage HUVECs. Recent studies indicate that glutaminolysis is crucial for the survival of senescent cells both *in vitro* and *in vivo*, as inhibition of glutaminolysis eliminates senescent cells and improves age-related organ dysfunction (184). Exploring the role of glutaminolysis in EC metabolism under various disease states may unveil novel druggable targets to regulate EC senescence.

Glucose and glutamine are both important fuel sources for the TCA cycle in mammalian cells. Glucose serves as the primary energy source for many cell types, while glutamine plays a crucial role as a carbon and nitrogen source for amino acid and nucleotide synthesis. The transporters SLC7A5 and SLC38A1 play critical roles in maintaining proper intracellular glutamine homeostasis. SLCA5 is involved in maintaining appropriate intracellular glutamine by facilitating glutamine efflux when levels are sufficient. However, overexpression of SLC7A5 has been associated with increased glutamine efflux, leading to decreased intracellular glutamine levels. This metabolic alteration is associated with an increased reliance on glucose metabolism and activation of oncogenic pathways, promoting tumor growth (299). SLC38A1, closely related to SLC38A9, is responsible for transporting glutamine into lysosomes in a pH-dependent manner. This transport process may contribute to a decrease in cytosolic glutamine levels. Lysosomes are organelles involved in macromolecule degradation, and their proper function relies on the availability of amino acids for the synthesis of new lysosomal proteins and enzymes (300). The relationship between the oncogene c-myc and glutamine transporters SLC7A5/SLC7A11 has been investigated in cancer research. C-myc has been found to upregulate the expression of SLC7A5 and SLC7A11, promoting tumor growth by enhancing amino acid update (301–303). On the other hand, studies have shown that the expression of the glutamate/cystine antiporter SLC7A11 in cancer cells is associated with reduced dependence on glutamine metabolism and increased sensitivity to glucose deprivation (304). While the function of glutamine transporters has been extensively studied in cancer cells, their precise role in ECs exposed to blood flow remains uncertain. It has been suggested that glutamine transporters, including SLC7A5, SLC7A11, and SLC38A1, may facilitate glutamine efflux, leading to a decrease in cytosolic glutamine levels (303, 305). Furthermore, SLC38A1 can transport glutamine into the lysosome, further impacting cytosolic glutamine levels (272, 274, 306, 307). Further research is necessary to understand the specific role of glutamine transporters in ECs under conditions of blood flow.

#### 7.5.4. Metabolic characteristics of senescent ECs and the SASP

The metabolic characteristics of senescent ECs and their connection to the SASP are still not fully understood. Among the sirtuin family, SIRT3, a mitochondrial member, has emerged as a key regulator of cellular aging. Through its NAD-dependent deacetylase activity, SIRT3 plays a role in regulating mitochondrial metabolic pathways, including FAO. It enhances the activity of enzymes such as PDH and acyl-CoA dehydrogenase, facilitating substrate entry into the TCA cycle. However, the precise role of SIRT3 in TCA cycle regulation is still a matter of debate. Depletion of SIRT3 has been associated with reduced human lifespan, like other sirtuin family members. Strategies aimed at upregulating SIRT3 in ECs have shown increased protection against SIPS by promoting the deacetylation of FoxO3. However, it is important to note that ECs exhibit distinct metabolic alterations during senescence compared to other cell types. For instance, the upregulation of glycolysis observed in senescent HDFs is not consistently observed in HUVECs. Conversely, recent findings suggest a senescence-associated decrease in EC glycolysis, mediated by reduced activity of PFKFB3. Overexpression of nuclear factor erythroid 2-related factor 2 (NRF2) can reverse this trend in ECs without affecting the senescence process. In another study focusing on RS in ECs, a slight increase in aerobic glycolysis was observed. This metabolic shift was mediated by the NAMPT/SIRT1/FoxO1 pathway and had a protective effect by limiting ROS production.

## 8. Discussion and future directions

The regulation of cellular senescence through metabolic alterations has been extensively studied; however, there are still many aspects that required further exploration, particularly regarding the development of EC senescence. The impact of metabolic changes on EC senescence is a fascinating area that remains incompletely understood (100). In quiescent ECs, glycolysis serves as the primary source of cellular ATP, contributing up to 85% of the total ATP content (273, 278, 280, 281). Mitochondrial function in ECs is primarily associated with biosynthesis and signaling rather than ATP production (286, 287, 308, 309). However, senescent ECs exhibit a distinct metabolic shift, diverging from other cell types that rely more heavily on glycolysis for energy production during senescence. Some studies have reported a decline in glycolysis in senescent ECs (298, 310), while others have observed a reduction in mitochondria-mediated OXPHOS (311–313). This raises the question of the energy source that fuels senescence and the SASP in ECs.

Further investigation is needed to unravel the underlying mechanisms and identify potential therapeutic targets in the field of EC senescence. Understanding the distinct metabolic regulations governing RS and SIPS is particularly important. Additionally, the roles of other amino acids, such as serine, asparagine, and aspartate, in EC metabolism during vessel sprouting require further exploration. Investigating the involvement of NAD^+^ metabolism and the role of the SIP in regulating EC senescence are areas that require attention. Moreover, studying the impact of blood flow on the metabolic regulation of EC senescence, as well as exploring differential glutamine metabolism between ECs and cancer cells, hold significant research potential. These investigations will contribute to a deeper understanding of the metabolic regulation of EC senescence and its implications in various physiological and pathological processes, including the effects of cancer therapies.

Blood flow patterns play a significant role in influencing disease progression *in vivo*, although the underlying biological mechanisms are not yet fully elucidated. Vascular ECs exposed to disturbed flow (D-flow) at bifurcation areas, but not laminar flow (L-flow), have been found to contribute to the development of atherosclerosis (314–316). D-flow generated at the distal regions of obstructive plaque further promotes atherosclerosis (317, 318). Although EC metabolism is believed to play a role in regulating EC function, the mechanisms by which different blood flow patterns regulate EC metabolism are still unclear. Doddaballapur et al. (319) reported that L-flow inhibits EC glycolysis by upregulating KLF2, but their study only showed slight inhibition of glucose uptake by L-flow. All other metabolite studies were performed under KLF2 overexpression and not specifically under L-flow conditions. Therefore, it is difficult to conclude definitively that L-flow inhibits glycolysis. In another study, Hong et al. (320) suggested that D-flow increases glycolysis, while L-flow increases FAO and OXPHOS. However, their conclusions were based on in silico transcriptome analysis of RNA-Seq data sets from two published papers. These analyses revealed an increase in mRNA expressions related to glycolysis under D-flow compared to L-flow in ECs. However, the authors did not provide metabolite profiling or tracing data, nor did they compare the effects of each flow pattern to a static (no flow) condition (319, 320). Therefore, the specific effects of blood flow patterns on EC metabolism remain unclear. Further studies incorporating comprehensive metabolite profiling and direct comparisons with static conditions are needed to elucidate the pattern-dependent effects of blood flow on EC metabolism.

The deSUMOylation enzyme SENP2, which contains multiple nuclear localization/export signals and exhibits nucleocytoplasmic shuttling activity, plays a crucial role in regulating the SUMOylation of various proteins, including ERK5, p53, FAK, and MAGI1 (321–327). Our previously published study demonstrated that D-flow activates p90RSK, leading to the phosphorylation of SENP2 at T368. This phosphorylation event enhances the SUMOylation of ERK5 and p53, ultimately triggering EC inflammation and apoptosis. These processes contribute to EC dysfunction and the development of atherosclerosis (247, 321–323, 327–330). Additionally, our study revealed that L-flow (12 dyn/cm^2^ by cones with a smooth surface) inhibits the SUMOylation of ERK5 and p53 without affecting SENP2 T386 phosphorylation (321, 322, 331). These molecules have the potential to serve as targets for the metabolic regulation of EC senescence.

Metabolic pathways play a crucial role in providing energy to macrophages and contribute to the regulation of their phenotype and function. Proinflammatory macrophages, which are activated by lipopolysaccharide and interferon-γ, exhibit enhanced glycolytic metabolism and impaired OXPHOS. This metabolic profile allows them to effectively mediate host defense mechanisms (332). Conversely, anti-inflammatory macrophages, activated by interleukin-4 (IL-4), primarily rely on OXPHOS for ATP synthesis. These macrophages are involved in processes such as wound healing and Th2-mediated immune responses (332, 333). The understanding of EC metabolism in this process is still limited and requires further investigation. Furthermore, the specific role of glutamine in energy production and proliferation has been extensively studied in cancer cells (37, 334–338). However, its precise role in ECs, particularly in response to blood flow, remains unclear. Further research is needed to elucidate the involvement of EC metabolism and the impact of glutamine in these processes.

The utilization of metabolomic tracing and profiling, in conjunction with spatial transcriptome analysis, has emerged as a valuable approach for investigating metabolic regulation (339). Metabolomic tracing involves the introduction of stable isotopes into metabolites, enabling the tracking of their metabolic fate and flux (340). By tracing isotopically labeled substrates, such as glucose or glutamine, we can discern how these metabolites are utilized in diverse metabolic pathways, providing a comprehensive understanding of cellular metabolism (341–345). Metabolomic profiling, on the other hand, entails the comprehensive analysis of metabolites present in a biological sample. Techniques such as mass spectrometry or nuclear magnetic resonance spectroscopy are employed to quantify and identify metabolites, generating a metabolic profile. This profiling approach facilitates the characterization of metabolic changes associated with specific conditions or perturbations, offering insights into metabolic dysregulation and potential therapeutic targets. Spatial transcriptome analysis, a powerful technique, combines spatial information with transcriptomic profiling. It allows for the simultaneous measurement of gene expression and spatial location within tissues or organs. By integrating spatial transcriptomics with metabolomic data, we can uncover spatially distinct metabolic pathways and identify cell-specific metabolic activities within complex biological systems (346, 347). This integration enables the discovery of novel metabolic regulations in specific cell types or microenvironments, illuminating tissue-specific metabolic adaptations and their implications in various physiological and pathological processes. The integration of metabolomic tracing, profiling, and spatial transcriptome analysis presents a comprehensive approach to investigating metabolic regulation. It facilitates the identification of key metabolic pathways, their spatial distribution, and their association with specific cellular functions or phenotypes (348). This integrated approach holds great promise in unraveling the complex network of metabolic regulations and their roles in health and disease.
